# Tumor lactate metabolism shapes immune suppression and therapeutic resistance revealed by integrative multi-omics and digital pathology

**DOI:** 10.3389/fimmu.2026.1797798

**Published:** 2026-03-26

**Authors:** Bohai Feng, Yongwu Zhu, Zheng Zhang, Yu Wang, Patrick J. Schuler, Jochen Hess

**Affiliations:** 1 Artificial Intelligence Research Center, Zhejiang Key Laboratory of Medical Epigenetics, Department of Cell Biology, School of Basic Medical Sciences, Hangzhou Normal University, Hangzhou, China; 2 Department of Otorhinolaryngology, Head and Neck Surgery, University Hospital Heidelberg, Heidelberg, Germany; 3 Jinshiyan Biotechnology, Yongkang, China; 4 Department of Stomatology, The First People’s Hospital of Yongkang, Yongkang, Zhejiang, China; 5 Department of Pathology, The Second Affiliated Hospital, Zhejiang University School of Medicine, Hangzhou, China; 6 Zhejiang Key Laboratory of Medical Epigenetics, Department of Biochemistry and Molecular Biology, School of Basic Medical Sciences, Hangzhou Normal University, Hangzhou, China; 7 Division Radiooncology/Radiobiology, German Cancer Research Center (DKFZ), Heidelberg, Germany

**Keywords:** digital biomarker, digital pathology, head and neck squamous cell carcinoma, lactate metabolism, metabolic phenotyping

## Abstract

**Background:**

Lactate metabolism is a hallmark of cancer metabolic reprogramming, shaping tumor immunity and therapeutic resistance, yet clinically accessible and low-cost methods to assess intratumoral lactate activity remain limited.

**Methods:**

We curated a lactate-related 59-gene signature and characterized its biological and clinical relevance across TCGA, GEO, and single-cell RNA-seq datasets. By integrating multi-omic, spatial, and computational analyses, we linked lactate metabolism to the tumor microenvironment and developed a deep learning framework to infer lactate metabolic states directly from routine H&E whole-slide images.

**Results:**

High lactate activity (LAC_H) was associated with enhanced tumor proliferation, suppressed immune infiltration, and poor response to both immunotherapy and radiotherapy in HNSCC. The pathology-based model achieved robust performance in distinguishing LAC_H from LAC_L tumors (AUC = 0.73–0.82 in HNSCC) and demonstrated strong generalizability across 12 TCGA cancer types (AUC = 0.78–0.89). Importantly, external validation in an independent real-world SAZHU-HNSCC cohort confirmed that model-predicted LAC_H tumors exhibited significantly increased protein expression of LDHA and MCT1 by immunohistochemistry, supporting the biological validity of the digital lactate biomarker.

**Conclusions:**

This study integrates multi-omics and digital pathology to infer tumor lactate metabolism from routine histology, providing a scalable and clinically practical digital biomarker for metabolism-informed precision oncology.

## 1 Introduction

The metabolic reprogramming of tumor cells is now recognized as a hallmark of cancer, enabling adaptation to microenvironmental stresses, supporting biomass accumulation, and fostering therapeutic resistance (1). Lactate secretion is a well-established metabolic hallmark exemplifying the Warburg effect. This phenomenon occurs when cancer cells prioritize glycolysis for energy production even in the presence of oxygen. The result is excessive lactate accumulation, which functions not as a mere metabolic byproduct, but rather as a dynamic modulator of the tumor microenvironment (TME) and malignant progression (2, 3). Importantly, lactate is also a major contributor to TME acidity, and this acidification is closely linked to immune suppression and resistance to immunotherapy (4). Recent reviews underscore that elevated lactate levels within tumors act as key immunomodulators by inhibiting cytotoxic T cell and natural killer cell activity, promoting regulatory T cell (Treg) expansion, and polarizing tumor-associated macrophages toward an M2-like phenotype (5–7).

Despite advances in surgery, radiotherapy, and immune checkpoint inhibitors (ICIs), the prognosis in head and neck squamous cell carcinoma (HNSCC) remains unsatisfactory due to pronounced tumor heterogeneity, immune escape, and metabolic adaptation (8). Emerging evidence increasingly implicates lactate metabolism as a central mediator of these resistance mechanisms (6, 9, 10). Beyond these metabolic and immunological effects, lactate serves as a substrate for histone and DNA modifications, a process termed “lactylation”, which epigenetically reprograms both immune and stromal cells in the TME (11, 12). Together, these findings suggest that lactate metabolism contributes to both metabolic adaptation and immune resistance in HNSCC. Key enzymes and transporters involved in lactate production and transport, as well as regulators of lactylation, may represent potential therapeutic targets and biomarkers to guide rational combination strategies in metabolically reprogrammed HNSCC (7).

Recent studies have begun to elucidate the metabolic and immunologic dimensions of lactate biology in HNSCC; however, its translational implications remain largely undefined. The discrepancy can be attributed to the insufficient characterization of the interplay between lactate-associated transcriptional states, cellular heterogeneity, and clinical phenotypes such as histopathology and therapy response. To address this gap, we curated a gene set capturing key elements of lactate metabolism and integrated multi-omic, spatial, and computational approaches to delineate its clinical and biological relevance in HNSCC.

Accordingly, this study aims to establish a molecular framework of lactate metabolism and examine its associations across clinical, immunological, and spatial dimensions. By integrating multi-omics and image-based analyses, we sought to connect metabolic activity with tumor architecture and pathology, and to develop a scalable, data-driven model capable of inferring metabolic states from routine histology. Collectively, these efforts aspire to advance precision oncology through low-cost, readily deployable tools that enable metabolism-informed patient stratification and therapeutic guidance.

## 2 Materials and methods

### 2.1 Gene signature collection

We collected lactate-related genes from the GeneCards database (https://www.genecards.org/) by searching with the keyword “Lactate”. The associated parameters, including relevance score and other annotations, were also downloaded from GeneCards (13). A total of 59 protein-coding genes with a GeneCards Relevance score >= 10 were retained, representing all entries meeting these criteria and aiming to capture a broad lactate-associated transcriptional landscape.

The immune and stromal signature framework was constructed using gene sets originally defined in Bagaev et al. (14) which systematically characterized TME components across solid tumors.

The T cell exhaustion gene set was originally defined in Zhang et al (15), and the M1 and M2 macrophage gene set was originally defined in Sullivan et al (16).

The Histology-Based Immune Features framework was constructed using gene sets originally defined by Cha et al. (17), encompassing tumor-infiltrating lymphocytes (TILs), plasma cell infiltration, tertiary lymphoid structures (TLS), and high-endothelial venule (HEV)-associated lymphoid aggregates (HALA.TLS), representing HEV-associated lymphoid structures within or surrounding tumor tissues. For TLS and HALA.TLS, scores were derived for total, intratumoral, peritumoral, and nontumoral regions to capture the spatially resolved immune architecture.

### 2.2 Protein-protein interaction analysis

Protein-protein interaction (PPI) analysis was conducted using the Search Tool for the Retrieval of Interacting Genes/Proteins (STRING) online database (18).

### 2.3 Acquisition of TCGA data

In November 2023, we obtained clinical data, gene expression profiles and diagnostic H&E-stained whole slide images (WSIs) for the TCGA cohorts from the Genomic Data Commons (GDC) portal (https://portal.gdc.cancer.gov/).

### 2.4 Acquisition of other bulk-seq data

In November 2024, the GSE179730 (19) dataset was retrieved from the NCBI Gene Expression Omnibus (GEO; https://www.ncbi.nlm.nih.gov/geo/). In addition, the melanoma cohort [PRJEB23709 (20) and phs000452 (21)] was accessed from the database of Genotypes and Phenotypes (dbGaP; https://www.ncbi.nlm.nih.gov/gap/).

### 2.5 Ligand-receptor interaction level calculation

Ligand-receptor (LR) levels for TCGA tumors were quantified using the BulkSignalR package (22).

### 2.6 Differential gene expression analysis

Differentially expressed genes between the lactate high (LAC_H) and lactate low (LAC_L) groups were identified using the EdgeR package (23).

### 2.7 Survival analysis

Kaplan-Meier survival analysis as well as univariate and multivariate Cox regression models were performed using the survival and survminer packages within the software R. Optimal cutoff values were determined with the maxstat package, utilizing the parameters method = “LogRank”, minprop = 0.2, maxprop = 0.8.

### 2.8 Gene set enrichment analysis

The Gene Set Enrichment Analysis (GSEA) was performed to identify significantly enriched pathways, with normalized enrichment scores and statistical significance calculated using the fgsea package.

### 2.9 Single sample gene set enrichment analysis

Enrichment scores for bulk RNA-seq data were calculated using single sample Gene Set Enrichment Analysis (ssGSEA) implemented in the GSVA package.

### 2.10 Single-cell transcriptomic integration

Detailed preprocessing, integration, clustering, malignant cell identification, and bulk single-cell deconvolution procedures for the scRNA-seq datasets are described in the **Supplementary Methods**.

### 2.11 SAZHU-HNSCC cohort and sample collection

The SAZHU-HNSCC cohort consisted of digitized H&E-stained WSIs from untreated surgical specimens of HNSCC patients, collected at the Department of Pathology, The Second Affiliated Hospital, Zhejiang University School of Medicine, between June 2024 and June 2025. All cases were histopathologically confirmed as HNSCC by experienced pathologists. FFPE tissues from selected patients were retrieved for immunohistochemical (IHC) staining of lactate metabolism-associated markers, including LDHA and MCT1. Corresponding digitized H&E-stained WSIs were archived for downstream histopathological analysis and deep learning-based lactate metabolism prediction. Clinical and pathological information was obtained from electronic medical records when available.

### 2.12 WSI acquisition and preprocessing

To manage the large volume of WSIs, we implemented a standardized preprocessing pipeline. Each slide was divided into non-overlapping 512 × 512-pixel tiles at 20-fold magnification. White background areas were excluded based on color saturation using a brightness threshold of 216, effectively removing low-information regions. Color normalization was subsequently applied using the Vahadane method to correct for staining variability (24).

### 2.13 Pathology morphological feature extraction and representation learning

To extract patch-level feature representations, the classification head of the CTransPath model (https://github.com/Xiyue-Wang/TransPath) was removed and replaced with an identity layer, enabling the penultimate layer to output 768-dimensional embeddings (25). Inference was performed in batches of 256 to improve computational efficiency. The extracted features, along with corresponding patch-level identifiers, were stored in compressed HDF5 (.h5) format on a per-slide basis.

To generate slide-level representations from patch embeddings, we implemented a customized transformer-based aggregation model (https://github.com/peng-lab/HistoBistro/tree/main) (26) designed to capture global contextual relationships among spatially distributed image regions. Specifically, 768-dimensional patch embeddings extracted from CTransPath were linearly projected to 512 dimensions via a fully connected layer with ReLU activation. A learnable classification token ([CLS]) was prepended to each sequence to represent the whole-slide context. The resulting sequence was processed through two transformer layers, each consisting of multi-head self-attention and feedforward networks with LayerNorm and residual connections. Self-attention was implemented using four attention heads with 128-dimensional subspaces, enabling the integration of long-range morphological dependencies. The final 512-dimensional slide-level embedding was derived from the [CLS] token output of the last transformer block. To reduce dimensionality and minimize redundancy, we applied PCA and retained the top 128 principal components as the final deep learning-derived features for each WSI.

### 2.14 Cross-omics analysis and modeling stratified by distinguish lactate levels from pathology imaging data

Each patient was represented by a 512-dimensional WSI embeddings were subsequently reduced to 128 dimensions using PCA, providing a compact representation of tissue morphology for downstream modeling. To refine the feature set, we applied LASSO regression. The optimal regularization parameter (alpha) was determined via cross-validation to minimize prediction error while encouraging model sparsity. Features with non-zero coefficients were retained, thereby selecting the most informative variables and enhancing model robustness. Subsequently, we trained four machine learning classifiers, namely XGBoost, Gradient Boosting, LightGBM, and Support Vector Machine (SVM) models, to predict patient risk groups based on the selected multimodal features. Model performance was evaluated across cancer types using the area under the receiver operating characteristic curve (AUROC) calculated from predicted class probabilities, decision curve analysis (DCA), and sample-level prediction score distributions to assess predictive accuracy and clinical utility. Threshold-dependent metrics, including accuracy, sensitivity, specificity, positive predictive value (PPV), and negative predictive value (NPV), were derived using probability cutoffs optimized by the Youden index. Confusion matrices were generated using a fixed probability threshold of 0.5 to provide a consistent reference for binary classification.

### 2.15 Integrated model-predicted lactate score

An integrated lactate prediction score was constructed by aggregating outputs from four machine learning models: XGBoost, Gradient Boosting, LightGBM, and SVM. Each model generated a slide-level predicted probability for the LAC_high class based on deep learning–extracted histopathological features. The integrated lactate score for each sample was calculated as the unweighted sum of the predicted class probabilities from the four models, yielding a continuous score ranging from 0 to 4. This ensemble-based score was subsequently used for SAZHU-HNSCC patient stratification and downstream analyses.

### 2.16 Immunohistochemistry and H-score evaluation

FFPE tissue sections from selected patients were deparaffinized, rehydrated, and subjected to antigen retrieval in EDTA buffer (pH 9.0). Endogenous peroxidase activity was blocked with 3% hydrogen peroxide, followed by blocking with bovine serum albumin. Sections were incubated overnight at 4 °C with primary antibodies against LDHA (1:500; Proteintech, Cat#19987-1-AP) and MCT1 (1:600; Proteintech, Cat#20139-1-AP), followed by incubation with HRP-conjugated secondary antibodies (HaoKebio, Cat#HKI0026). Immunoreactivity was visualized using DAB chromogen and counterstained with hematoxylin (HaoKebio, Cat#HK2053).

Digitized IHC slides were quantitatively analyzed using Visiopharm software. H-scores were calculated exclusively within annotated tumor regions. The H-score was computed as H-Score = ∑(pi×i)= (percentage of weak intensity cells ×1)+ (percentage of moderate intensity cells ×2)+ (percentage of strong intensity cells ×3). H-scores ranged from 0 to 300, with higher values indicating stronger protein expression.

### 2.17 Software and statistics

The study employed a range of software tools, including custom Python code written in Python v.3.7.12. The Python packages used in the analysis included Pandas v.1.2.4, NumPy v.1.20.2, Transformers v.4.51.3, PyTorch v.1.8.0, OpenSlide v.1.2.0, Seaborn v.0.11.1, Matplotlib v.3.4.2, SciPy v.1.7.3, and scikit-learn v.1.0.2.

Additionally, custom R code was written in R v.4.4.0, utilizing various R packages such as Seurat v.4.4.0, harmony v.1.2.0, CellChat v2.1.2, msigdbr v.7.5.1, GSVA v.1.52.0, fgsea v.1.30.0, clusterProfiler v.4.12.0, ggplot2 v.3.5.1, infercnv v.1.20.0, BayesPrism v.2.2.2, BulkSignalR v.0.0.9, oncopredict v.1.2, maftools v.2.20.0, maxstat v.0.7-25, TCGAbiolinks v.2.32.0, survminer v.0.4.9, randomForestSRC v.3.3.1, and randomSurvivalForest v.3.6.4, all run within the R 4.4.0 environment. The convaq v.0.1.3 package was run separately within the R 3.6.3 environment.

Group comparisons were conducted using Wilcoxon tests (with a significance level set at p = 0.05), and all analyses were performed using Jupyter Notebook and RStudio.

## 3 Results

### 3.1 Construction of the lactate-related gene set and its clinical and molecular characterization

**Figure 1:**
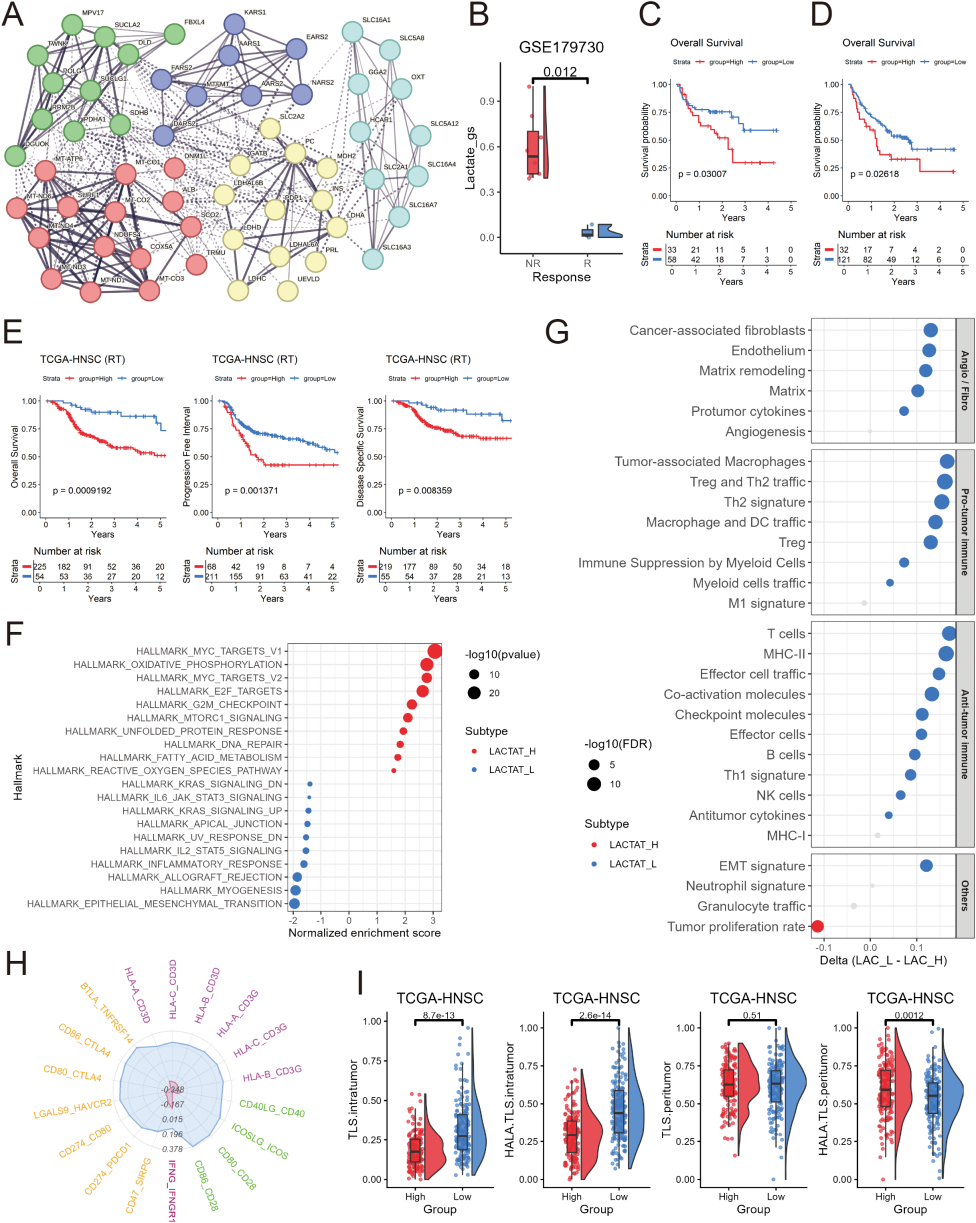
Construction and functional characterization of the lactate-related gene set. **(A)** Protein-protein interaction **(PPI)** network of genes included in the lactate-related gene set, showing functional connections and clustering modules. **(B)** Boxplot comparing ssGSEA scores of the lactate-related gene set between pre-treatment head and neck squamous cell carcinoma (HNSCC) tissues from patients with immune checkpoint inhibitor (ICI) response (R) and no response (NR) in the GSE179730 cohort. **(C, D)** Kaplan-Meier survival curves showing the predictive value of the lactate-related gene set score for overall survival in melanoma cohorts PRJEB23709 **(C)** and phs000452 **(D)** treated with ICI therapy. Patients were divided into high and low groups based on optimal cut-off values. **(E)** Kaplan-Meier survival analysis demonstrating that the lactate-related gene set score predicts overall survival, progression-free interval, and disease-specific survival in HNSCC patients receiving adjuvant radiotherapy. **(F)** Dot plot summarizing the gene set enrichment analysis (GSEA) of Hallmark gene sets between the top 33% (LAC_H) and bottom 33% (LAC_L) tumors ranked by lactate-related ssGSEA scores. The dot size represents -log_10_(p-value), and the color indicates the direction of enrichment. **(G)** Dot plot showing differences in tumor microenvironment composition scores (from Bagaev et al. (14)) between LAC_H and LAC_L HNSCC tumors (Delta = LAC_L - LAC_H). The dot size indicates –log_10_(p-value), and the color denotes the direction of change. **(H)** Radar plot displaying enrichment scores of ICI-related ligand–receptor (LR) pairs between LAC_H and LAC_L HNSCC tumors. Blue represents scores in LAC_L tumors, and red represents LAC_H tumors. **(I)** Boxplots illustrating differences in intratumoral and peritumoral tertiary lymphoid structure (TLS) and high-endothelial venule–associated lymphoid aggregate (HALA.TLS) scores levels between LAC_H and LAC_L HNSCC tumors from the TCGA cohort.

The GeneCards database was first searched using the keyword “Lactate”, yielding a total of 7,599 entries (**Supplementary Table 1**). Among them, 59 protein-coding genes with a relevance score >= 10 were selected, representing genes most closely associated with lactate metabolism, and were defined as the lactate-related 59-gene set for further analysis. To investigate the functional organization and connectivity of these genes, a PPI network was constructed using the STRING database and applied K-means clustering to identify co-functional modules (**Figure 1A**). This analysis revealed five major clusters, each enriched in distinct metabolic or mitochondrial functions: Cluster 1 (red): Respiratory electron transport chain (15 genes); Cluster 2 (yellow): Pyruvate metabolism (13 genes); Cluster 3 (green): Primary mitochondrial disease (11 genes); Cluster 4 (cyan): Lactate transmembrane transporter activity (10 genes); and Cluster 5 (blue): Aminoacyl-tRNA biosynthesis (8 genes). LDHB and MLDHR were not included in the STRING database and were therefore excluded from the clustering analysis. These clusters highlight the diverse yet interconnected metabolic processes underpinning lactate biology, encompassing mitochondrial oxidative phosphorylation, pyruvate-lactate interconversion, and aminoacyl-tRNA formation. Moreover, the strong connectivity among clusters underscores the central role of lactate metabolism in regulating cellular energy homeostasis and biosynthetic plasticity.

Recent studies have demonstrated that elevated lactate metabolism fosters an immunosuppressive TME and correlates with resistance to ICIs (7, 27, 28). Consistent with these reports, the analysis of pre-treatment HNSCC samples from the GSE179730 cohort (19) revealed significantly higher lactate-related ssGSEA scores in non-responders compared with responders (**Figure 1B**). Similarly, in two independent melanoma immunotherapy cohorts [PRJEB23709 (20) and phs000452 (21)], patients with higher lactate-related ssGSEA scores exhibited markedly worse overall survival following ICI treatment (**Figures 1C, D**). Together, these findings indicate that enhanced lactate metabolic activity is associated with reduced efficacy of immunotherapy and unfavorable clinical outcomes across multiple tumor types.

Besides its impact on immune evasion, lactate metabolism has also been implicated in modulating tumor radiosensitivity (7, 29). Elevated lactate levels contribute to a hypoxic and reductive microenvironment that promotes DNA damage repair and limits radiation-induced oxidative stress, thereby conferring radioresistance (30). Accordingly, stratification of TCGA-HNSCC patients receiving adjuvant radiotherapy was performed based on their lactate-related ssGSEA scores, followed by evaluation of differences in clinical endpoints (**Figure 1E**). This analysis revealed that patients with higher lactate-related scores exhibited significantly worse overall survival (OS), progression-free interval (PFI), and disease-specific survival (DSS) compared with those with lower scores, indicating that elevated lactate metabolic activity is also associated with poor prognosis in HNSCC under radiotherapy treatment. Furthermore, univariate and multivariate Cox regression analyses confirmed the lactate-related gene set score as an independent prognostic factor in HNSCC patients treated with radiotherapy (**Supplementary Figures 1A–C**). In contrast, patients who did not receive radiotherapy exhibited no measurable prognostic differences (data not shown).

To further elucidate the underlying biological mechanisms associated with different lactate-related scores, we performed GSEA comparing the top 33% (LAC_H) and bottom 33% (LAC_L) tumors from TCGA-HNSCC ranked by lactate-related ssGSEA scores. Notably, tumors in the LAC_H group exhibited significantly higher proportion of T3–4 stage tumors indicating more extensive local tumor growth (**Supplementary Table 2**) . In contrast, LAC_L tumors showed a higher frequency of lymph node metastasis (N+ cases), suggesting distinct patterns of tumor progression and dissemination between the two metabolic states (**Supplementary Table 2**).

To investigate the molecular basis underlying the differences between LAC_H and LAC_L tumors, we performed DEG analysis (**Supplementary Table 3**), and subsequently conducted GSEA to identify the associated biological pathways. As shown in **Figure 1F**, the LAC_H tumors were significantly enriched for hallmark pathways related to MYC targets, oxidative phosphorylation, fatty acid metabolism, mTORC1 signaling, DNA repair, and cell-cycle progression, suggesting enhanced metabolic and proliferative activity. Importantly, the glycolysis pathway was also significantly enriched in LAC_H tumors (ranked at position 11; see in **Supplementary Table 4**). In contrast, the LAC_L tumors showed enrichment of immune-related and epithelial-mesenchymal transition pathways, such as IL6-JAK-STAT3 signaling, inflammatory response, and allograft rejection, suggesting a more immune-active phenotype with relatively lower enrichment of proliferation-related pathways compared to LAC_H tumors.

To further dissect the TME differences associated with lactate-related metabolic activity, we next performed tumor composition analysis based on the immune and stromal signature framework proposed by Bagaev et al (14). As shown in **Figure 1G**, LAC_H tumors displayed only higher tumor proliferation activity, without broad enhancement of other tumor-promoting components. In contrast, LAC_L tumors demonstrated increased infiltration of anti-tumor immune signatures-including T cells, NK cells, B cells, effector cell trafficking, and MHC-I expression, indicating a more immune-active microenvironment. Notably, LAC_L tumors also showed higher enrichment of stromal and pro-tumor-associated components, such as cancer-associated fibroblasts (CAFs), endothelial cells, matrix remodeling, and angiogenesis signatures. Together, these results suggest that LAC_L tumors exhibit a more active and dynamic TME, characterized by both enhanced immune activation and elevated stromal remodeling.

We next compared the enrichment scores of immune related LRs between the LAC_H and LAC_L groups to further characterize the immunoregulatory landscape. As shown in **Figure 1H**, LRs including multiple ICIs, MHC-I, co-stimulatory and IFNG displayed higher enrichment in the LAC_L group, indicating enhanced antigen presentation and T-cell activation potential. In contrast, the LAC_H group exhibited globally reduced LR activity across these pathways, consistent with a more immunosuppressed TME.

In line with these observations, intratumoral TLS and HALA.TLS scores were also significantly higher in the LAC_L group, further supporting the presence of a more active intratumoral immune milieu. Conversely, the LAC_H group showed elevated peritumoral HALA.TLS scores, suggesting that lymphoid aggregation in high-lactate tumors tends to be spatially restricted to the tumor periphery rather than forming within the immunosuppressed tumor core (**Figure 1I**).

### 3.2 Single-cell characterization of lactate-related gene set expression and cellular composition in HNSCC

**Figure 2:**
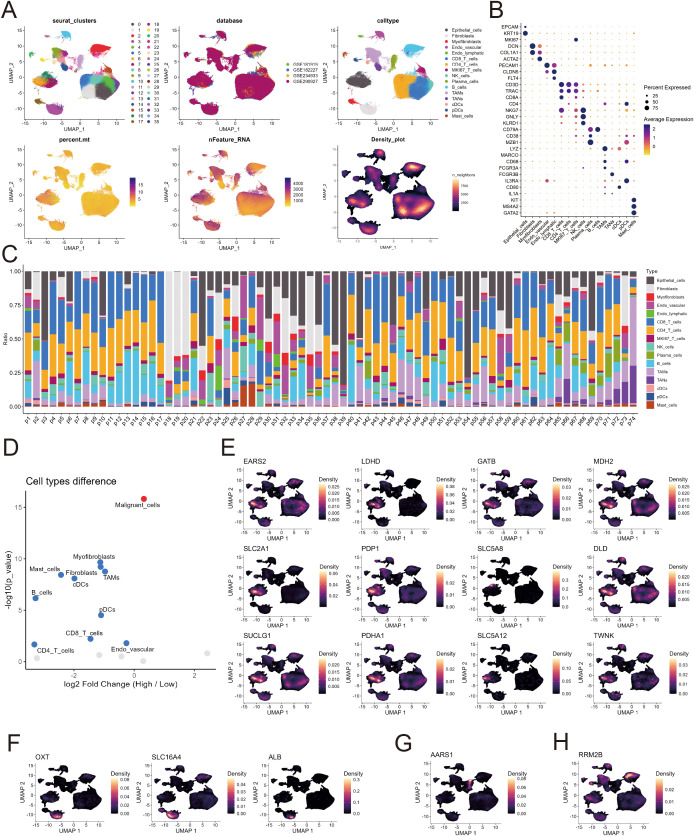
Single-cell characterization of lactate-related gene set localization and cellular composition in HNSCC. **(A)** UMAP plots showing single-cell transcriptomic profiles from 74 HNSCC patients across three public datasets. Cells are color-coded by Seurat clusters (upper left), dataset of origin (upper middle), annotated cell types (upper right), mitochondrial gene percentage (lower left), number of detected RNA features (lower middle), and cell density (lower right). **(B)** Dot plot displaying the expression of canonical marker genes across annotated cell types, illustrating distinct expression patterns defining each cell population. **(C)** Stacked bar plots showing the proportion of major cell types across individual HNSCC samples, revealing inter-sample heterogeneity in cellular composition. **(D)** Volcano plot showing differential abundance of cell types between LAC_H and LAC_L tumors inferred by BayesPrism-based cell type deconvolution. **(E–H)** Density plots illustrating the spatial localization and expression heterogeneity of genes from the lactate-related gene set at the single-cell level. Twelve genes are predominantly expressed in epithelial cells **(E)**, three genes are enriched in fibroblasts **(F)**, and one gene each shows higher expression in plasma cells **(G)** and B cells **(H)**.

To further elucidate the cellular localization and heterogeneity of the lactate-related gene set, we integrated single-cell RNA-seq data from four public HNSCC datasets, comprising a total of 74 patients (31–34). After batch correction and quality control, unsupervised UMAP clustering identified 36 major cell clusters across epithelial, immune, and stromal compartments (**Figure 2A**). Canonical marker expression confirmed the annotation of epithelial cells, fibroblasts, macrophages, T cells, B cells, and endothelial cells (**Figure 2B**). The proportional composition of these cell types varied markedly among patients (**Figure 2C**), reflecting strong intertumoral heterogeneity. To distinguish malignant from non-malignant epithelial populations, we further performed inferCNV analysis based on large-scale chromosomal copy number variations. Among a total of 39,830 epithelial cells, 33,892 were classified as malignant epithelial cells, whereas cells from clusters 6, 10, and 20 were identified as non-malignant and excluded from downstream analyses (**Supplementary Figure 2A**).

We next compared the relative abundance of each cell type between the LAC_H and LAC_L groups in TCGA-HNSC using BayesPrism-based deconvolution. Malignant cells were significantly enriched in the LAC_H tumors, whereas fibroblasts, macrophages, and multiple immune subsets were relatively depleted (**Figure 2D**). This suggests that high lactate metabolic activity is associated with an epithelial-dominant, less immune-infiltrated phenotype.

Among the 58 lactate-related genes analyzed (excluding MLDHR, which was not included in the single-cell datasets), 38 genes showed relatively high expression across multiple cell types (**Supplementary Figure 2B**), reflecting their broad metabolic involvement within the TME. In contrast, 12 genes were predominantly expressed in malignant epithelial cells (**Figure 2E**), underscoring their potential contribution to lactate metabolism in the epithelial compartment. Additionally, three genes were mainly enriched in fibroblasts (**Figure 2F**), while one gene exhibited preferential expression in either plasma cells (**Figure 2G**), B cells (**Figure 2H**), mast cells (**Supplementary Figure 2C**), plasmacytoid dendritic cells (pDCs) (**Supplementary Figure 2D**), or tumor-associated neutrophils (TANs) (**Supplementary Figure 1E**), respectively. These results highlight the extensive cellular diversity of the lactate metabolism.

### 3.3 Tumor morphological heterogeneity and deep learning-based histopathological prediction of lactate metabolism levels in HNSCC

**Figure 3:**
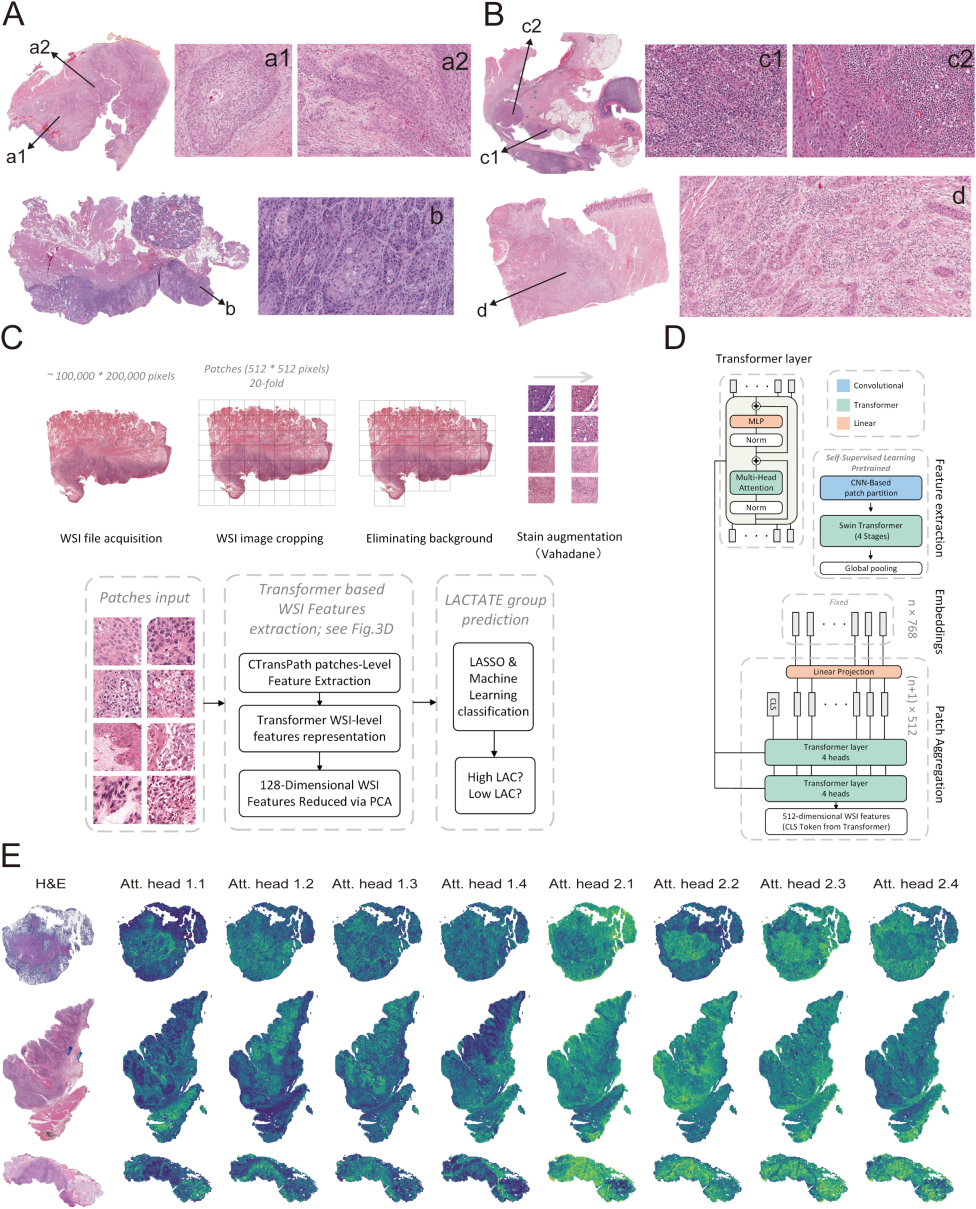
Prediction of LAC_H and LAC_L groups based on H&E-stained whole-slide images (WSIs). **(A, B)** Representative hematoxylin and eosin (H&E)-stained WSIs from the LAC_H **(A)** and LAC_L **(B)** groups, respectively. Enlarged regions (a1-a2, b, c1-c2, d) highlight differences in immune cell infiltration and tumor microenvironment architecture between groups. **(C)** Workflow illustrating the preprocessing and modeling of H&E-stained WSIs. Each image at 20x magnification was divided into 512 x 512-pixel patches, and background regions were excluded using saturation thresholds. Color normalization was applied using the Vahadane method. Patch-level features were extracted using the CTransPath model and aggregated into slide-level representations through a transformer-based architecture, followed by dimensionality reduction via principal component analysis (PCA) to obtain 128-dimensional WSI features. The resulting features were used in LASSO regression and machine learning classification to predict LAC_H or LAC_L group membership. **(D)** Schematic representation of the CTransPath-based patch feature extraction and transformer fusion architecture, showing convolutional, transformer, and linear projection layers used for hierarchical feature embedding and patch aggregation. **(E)** Attention heatmaps from eight attention heads, including four from the first and four from the second transformer layer.

Given the distinct cellular composition and immune microenvironmental features observed between the LAC_H and LAC_L groups, we next examined the morphological heterogeneity based on hematoxylin and eosin (H&E)-stained histopathological sections from TCGA-HNSCC samples. Notably, tumors in the LAC_H group appeared morphologically “cleaner”, with relatively homogeneous tumor nests and limited stromal or immune cell infiltration (**Figure 3A**). In contrast, LAC_L tumors exhibited a more “heterogeneous” architecture characterized by abundant immune and stromal infiltration (**Figure 3B**). In both **Figures 3A, B**, the upper inset shows a tumor in the tonsil, whereas the lower panel depicts a tumor in the oral cavity. These observations suggest that histopathological morphology may partially reflect underlying lactate metabolic states within the TME. To quantitatively assess the association between histopathological morphology and lactate metabolism, we developed a deep learning-based framework to predict lactate metabolic status directly from digitized H&E-stained WSIs (**Figure 3C**). We collected 500 TCGA-HNSCC cases from the GDC portal, including 450 diagnostic WSIs. Among these, 288 WSIs were selected for analysis, with the top and bottom 33% of cases ranked by lactate ssGSEA scores (165 each) defined as the LAC_High and LAC_Low groups, respectively. After preprocessing, a total of 2,971,293 normalized tissue patches were generated for downstream modeling. In total, 273 patients with available WSIs were randomly selected from a cohort of 330 patients and then randomly divided into training and testing cohorts at a 3:1 ratio. The training cohort consisted of 204 patients (216 WSIs), while the testing cohort included 69 patients (72 WSIs), corresponding to 2,287,206 and 684,087 image patches, respectively.

**Figure 4:**
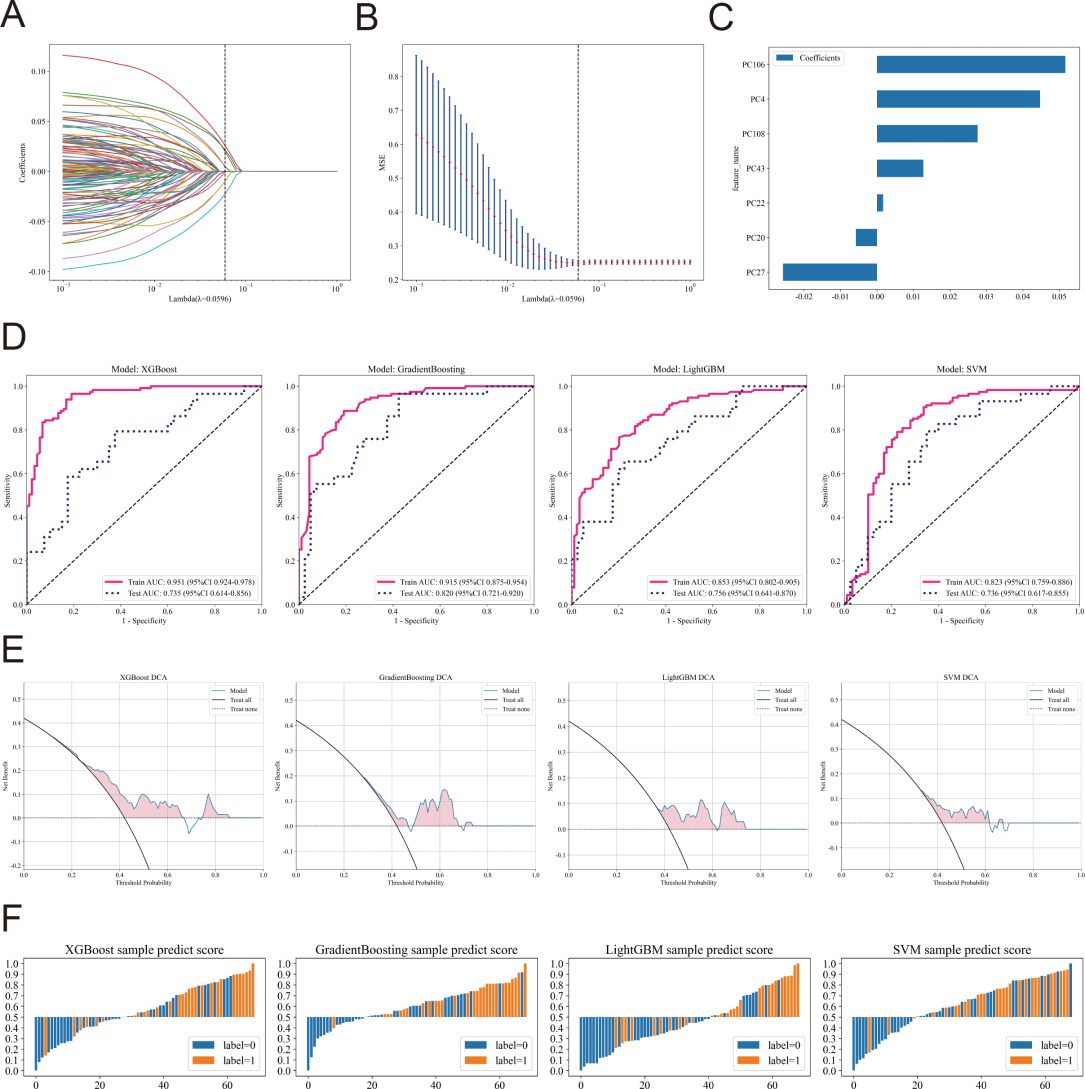
Prediction of LAC_H and LAC_L groups based on pathological machine learning models in HNSCC. **(A–C)** A total of 512-dimensional pathology-derived features were extracted from the transformer-based WSI model and reduced to 128 dimensions using PCA. LASSO regression identified seven key features for downstream modeling. **(D)** Receiver operating characteristic (ROC) curves and corresponding area under the curve (AUC) values of XGBoost, GradientBoosting, LightGBM, and support vector machine (SVM) models for LAC_H/LAC_L classification, evaluated on training and test cohorts. **(E)** Decision curve analysis (DCA) comparing the net clinical benefit of the four models, illustrating the potential utility of each model across varying threshold probabilities. **(F)** Sample-level prediction score distributions for XGBoost, GradientBoosting, LightGBM, and SVM models, showing separation between predicted LAC_H (label = 1) and LAC_L (label = 0) groups.

To model lactate-related histological phenotypes, patch-level features were first extracted from each WSI using the pretrained CTransPath network, and slide-level representations were then obtained through a transformer-based aggregation architecture. This resulted in 512-dimensional embeddings that captured global morphological patterns. These embeddings were subsequently compressed to 128 dimensions via PCA. LASSO regression was applied to identify the most informative features, which were finally used to train distinct models using XGBoost, Gradient Boosting, LightGBM, and SVM classifiers to distinguish LAC_H and LAC_L tumors (**Figures 4A–C**). In the test cohort, all models demonstrated strong predictive performance, with AUROC values ranging from 0.73 to 0.82, indicating robust discriminative capability (**Supplementary Table 5**; **Figure 4D**). DCA further supported the clinical utility of these models, while sample-level prediction score distributions revealed clear separation between the predicted LAC_H and LAC_L groups (**Figures 4E, F**). Collectively, these results highlight the potential of deep learning-derived histopathological features as quantitative surrogates for lactate metabolism in HNSCC.

### 3.4 Histopathological validation of deep learning–predicted lactate metabolism in SAZHU-HNSCC cohort

Based on the four machine learning models derived from deep learning–extracted histopathological features, we next generated an integrated lactate prediction score for each HNSCC sample. To validate whether the model-predicted lactate status reflected metabolic activity at the tissue level, we performed immunohistochemical (IHC) analysis of key lactate metabolism-associated proteins, including LDHA and MCT1, in an independent SAZHU-HNSCC cohort (**Supplementary Table 6**).

**Figure 5:**
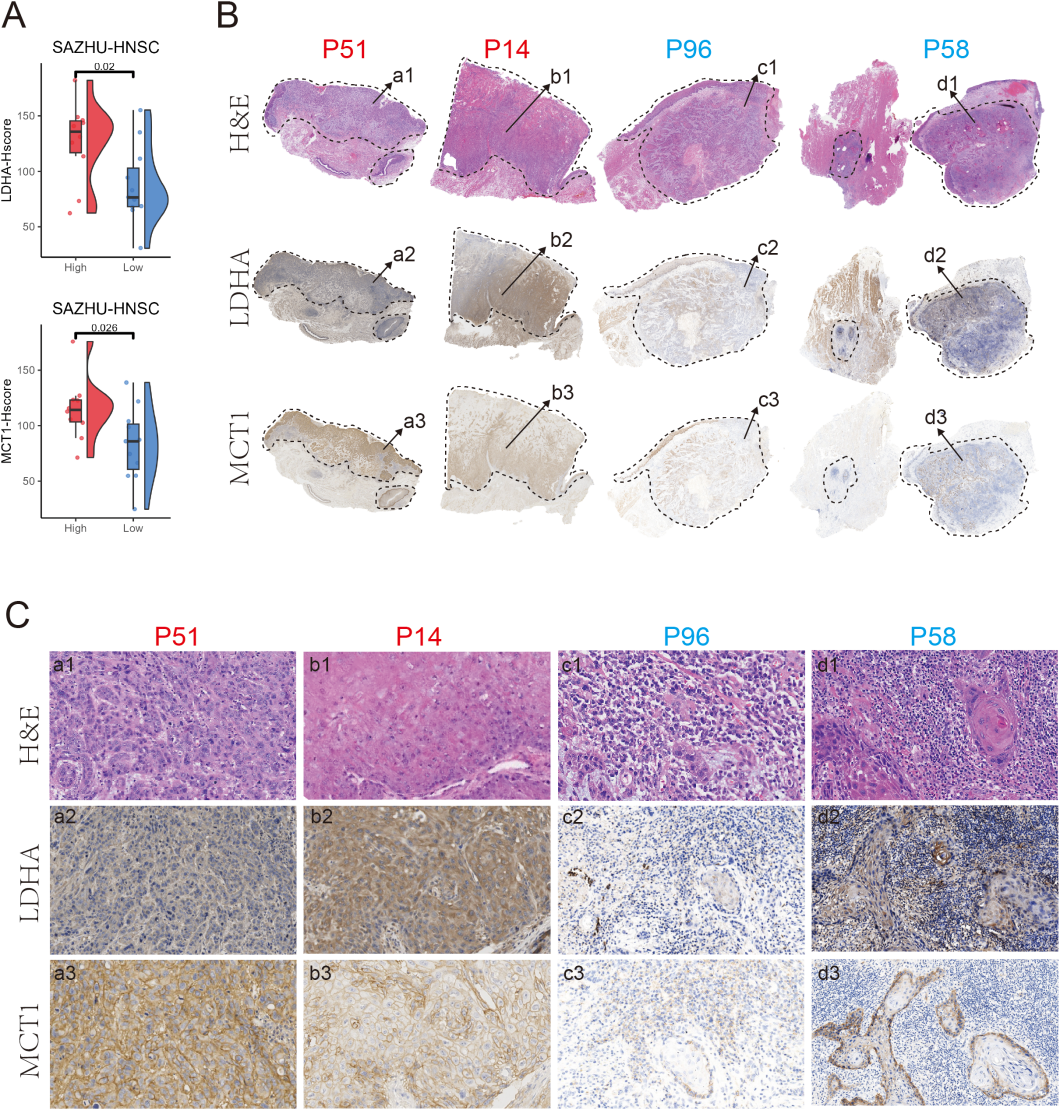
Association between deep learning-predicted lactate levels and LDHA and MCT1 IHC expression in SAZHU-HNSCC cohort. **(A)** Boxplots showing differences in LDHA and MCT1 immunohistochemical (IHC) H-scores within tumor regions between samples stratified by deep learning–predicted lactate levels in the SAZHU-HNSCC cohort. Patients were divided into the top 10% (high-lactate) and bottom 10% (low-lactate) groups. **(B)** Representative whole-slide images (WSIs) from the high- and low-lactate groups. From top to bottom, panels show hematoxylin and eosin (H&E) staining, LDHA IHC, and MCT1 IHC. High-lactate cases: P14 and P51 (red); low-lactate cases: P58 and P96 (blue). Dashed lines delineate tumor regions, and arrows indicate areas selected for higher-magnification analysis. **(C)** Representative high-magnification fields corresponding to the regions indicated in **(B)**. From top to bottom, panels show H&E staining, LDHA IHC, and MCT1 IHC.

A total of 110 patients in the SAZHU-HNSCC cohort were stratified according to the integrated model-predicted lactate scores, with the top 10% defined as the high-lactate group (11 cases; IHC available for 10 slides due to the unavailability of FFPE tissue from one patient, P107) and the bottom 10% as the low-lactate group (11 cases, 11 slides). Quantitative IHC analysis revealed that tumors in the high-lactate group exhibited significantly higher H-scores for both LDHA and MCT1 within tumor regions compared with those in the low-lactate group (**Figure 5A**), indicating enhanced lactate production and transport capacity in model-predicted lactate-high tumors.

Representative WSIs further illustrated distinct histopathological and metabolic features between the two groups (**Figure 5B**). High-lactate tumors (P14 and P51) exhibited relatively “clean” and compact tumor nests with markedly elevated LDHA and MCT1 staining levels. In contrast, low-lactate tumors (P58 and P96) showed increased immune cell infiltration accompanied by substantially lower LDHA and MCT1 expression. Consistent patterns were observed at higher magnification (**Figure 5C**), where tumor cells in the high-lactate group displayed strong LDHA and MCT1 immunoreactivity, whereas reduced staining intensity was evident in low-lactate tumors. These results demonstrate that lactate metabolic states inferred from H&E-based deep learning and machine learning models are concordant with protein-level expression of canonical lactate metabolism markers.

### 3.5 Pan-cancer characterization of lactate-associated microenvironmental and morphological differences

**Figure 6:**
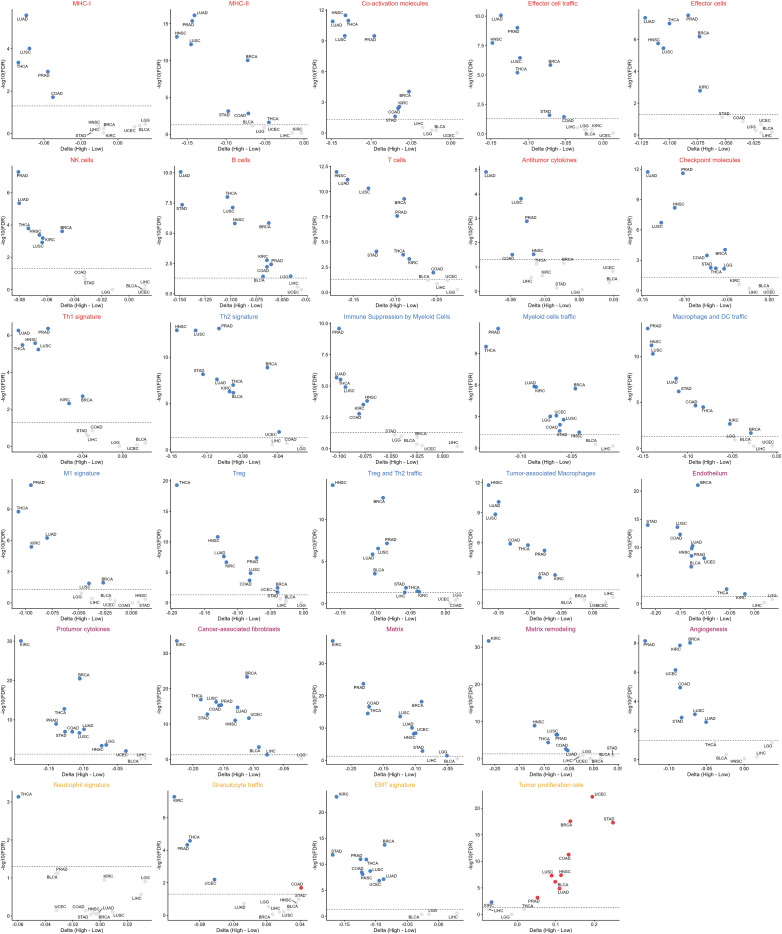
Pan-cancer differences in tumor microenvironment composition between LAC_H and LAC_L groups. Dot plots showing differences in tumor microenvironment composition scores between LAC_H and LAC_L tumors across multiple TCGA solid cancers. Delta represents the difference in mean ssGSEA enrichment scores between LAC_H and LAC_L tumors (mean score in LAC_H minus mean score in LAC_L). Each dot represents a cancer type, with the x-axis indicating the difference in signature score and the y-axis indicating statistical significance (−log10 p-value). Red dots denote cancer types with higher signature scores in the LAC_H group, and blue dots denote those with higher scores in the LAC_L group. The colors of the panel titles indicate the functional category of each signature (red, anti-tumor immune; blue, pro-tumor immune; brown, angiogenesis/fibroblast; yellow, other).

Tumor types with more than 200 cases and available diagnostic digital pathology data were selected, yielding 12 eligible pan-cancer cohorts: BLCA, BRCA, COAD, KIRC, LGG, LIHC, LUAD, LUSC, PRAD, STAD, THCA, and UCEC. Using the immune and stromal signature framework proposed by Bagaev et al. (14), we characterized tumor composition across these cohorts. Consistent with patterns observed in HNSCC, most cancer types showed that LAC_H tumors were defined primarily by increased tumor proliferation activity, whereas LAC_L tumors demonstrated both stronger immune activation and more extensive stromal remodeling, indicative of a more dynamically active TME (**Figure 6**).

We next evaluated LR interactions and found that multiple ICI-related pairs (**Supplementary Figure 3A**), co-stimulatory pairs (**Supplementary Figure 3B**), MHC-I–associated pairs (**Supplementary Figure 3C**), and the IFNG–IFNGR1 axis (**Supplementary Figure 3D**) were enriched in the LAC_L group across the majority of tumor types. In parallel, LAC_L tumors exhibited higher enrichment scores for T cell exhaustion signatures (**Supplementary Figure 4A**) as well as M1- and M2-like macrophage polarization programs (**Supplementary Figure 4B**) in most tumor types. LAC_L tumors also exhibited significantly higher intratumoral TLS and HALA.TLS scores, supporting the presence of a more active intratumoral immune milieu. Conversely, LAC_H tumors showed elevated peritumoral HALA.TLS scores (**Supplementary Figure 4C**).

**Figure 7:**
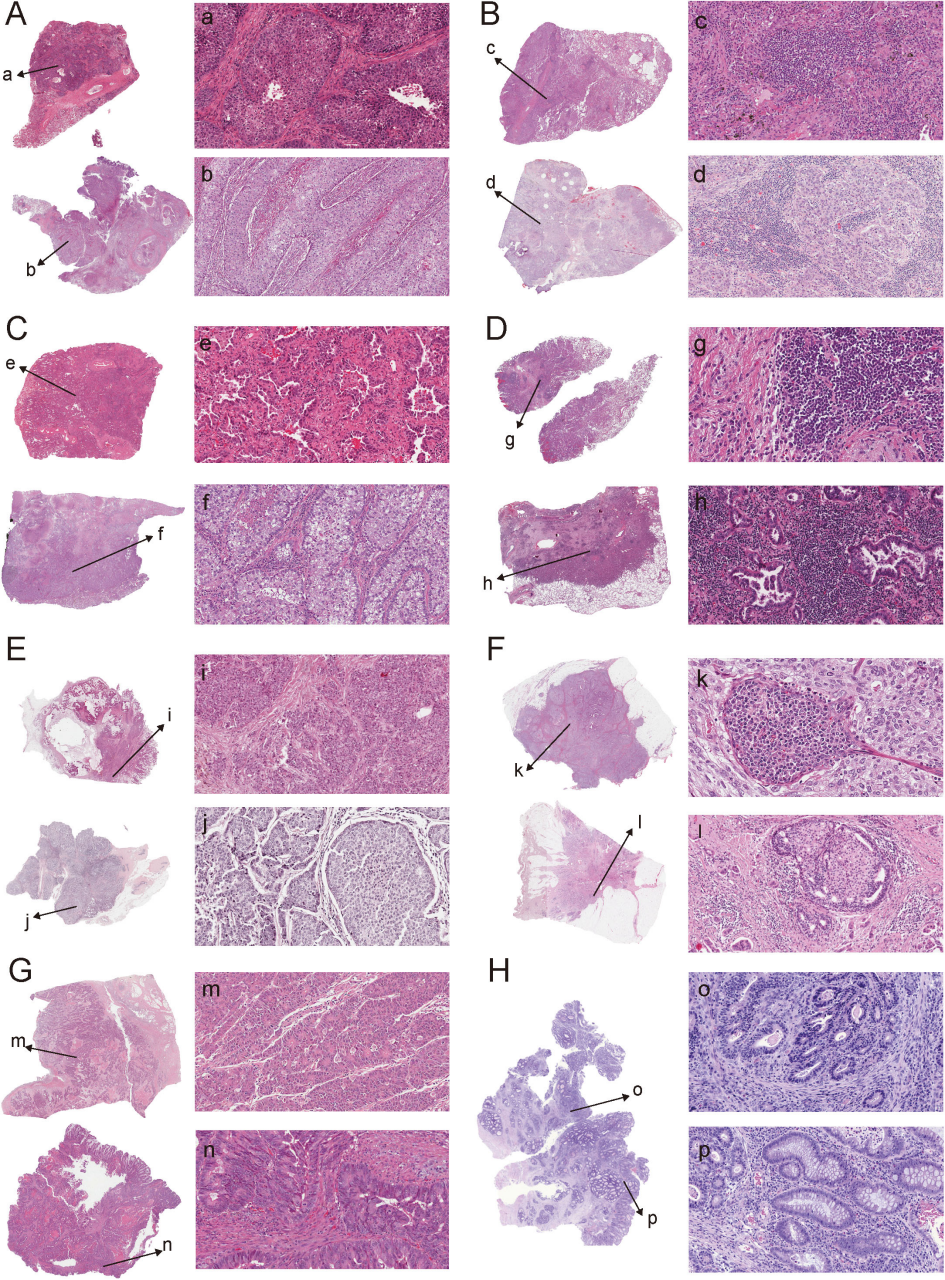
Representative H&E staining illustrating morphological differences between LAC_H and LAC_L tumors across four cancer types. LUSC: panel A shows LAC_H, panel B shows LAC_L. LUAD: panel C shows LAC_H, panel D shows LAC_L. BRCA: panel E shows LAC_H, panel F shows LAC_L. COAD: panel G shows LAC_H, panel H shows LAC_L. For each cancer type, whole-slide overviews (left) and corresponding high-magnification fields (right) are presented, with arrows indicating the regions selected for higher-resolution visualization.

Finally, H&E-stained sections revealed distinct morphological features across representative cancer types. In line with observations from HNSCC, LAC_H tumors displayed cleaner and more uniform architecture, whereas LAC_L tumors exhibited more heterogeneous structural patterns in LUSC (**Figures 7A, B**), LUAD (**Figures 7C, D**), BRCA (**Figures 7E, F**), and COAD (**Figures 7G, H**).

### 3.6 Pan-cancer generalization and validation of the pathology-based lactate metabolism model

**Figure 8:**
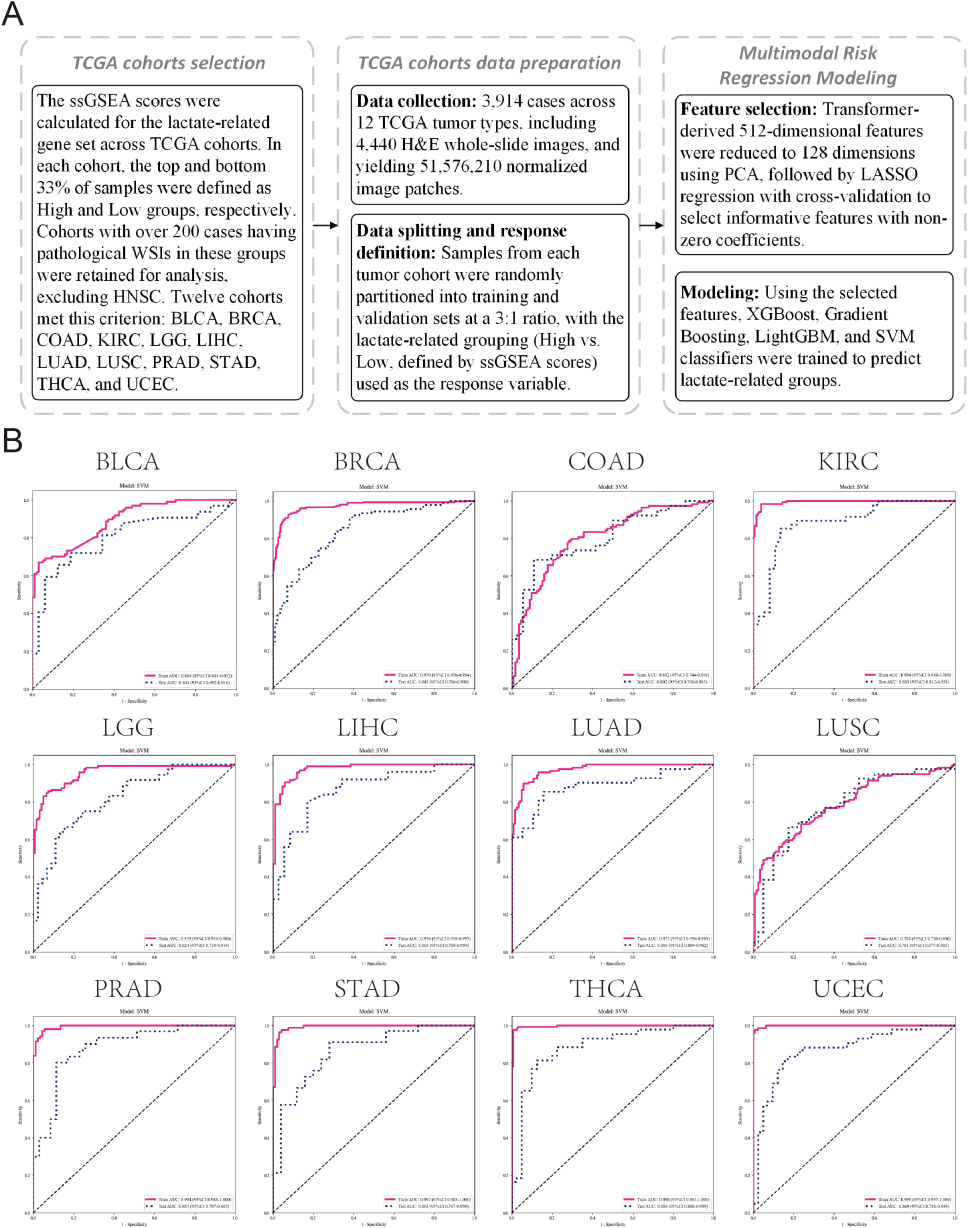
Analysis workflow and SVM model performance across TCGA cohorts (excluding HNSCC). **(A)** Schematic overview of the analysis pipeline for 12 TCGA tumor cohorts. ssGSEA scores for the lactate-related gene set were calculated, samples were classified into High and Low groups (top and bottom 33%), and cohorts with over 200 cases having pathological WSIs were retained. Transformer-derived 512-dimensional features from WSIs were reduced to 128 dimensions using PCA, followed by LASSO regression to select informative features. The selected features were used to train machine learning models for predicting lactate-related groups. **(B)** ROC curves and corresponding AUC values of SVM model for LAC_H/LAC_L classification, evaluated on training and test cohorts.

Given our earlier observations that lactate metabolic states are linked to distinct tumor microenvironmental features and pronounced differences in H&E morphology across multiple cancer types, we next investigated whether these lactate-associated phenotypes could be detected by a deep learning-based pathology model in a pan-cancer setting. To this end, we extended the analysis to 12 additional solid tumor cohorts from TCGA. In total, 3,914 cases across 12 tumor types were included, comprising 4,440 H&E-stained WSIs and yielding 51,576,210 normalized image patches (**Figures 8A**; **Supplementary Table 7**). For each cohort, ssGSEA scores were calculated using the lactate-related gene set, and samples were classified into LAC_H and LAC_L groups according to the top and bottom 33% of scores. Based on transformer-derived WSI features, multiple machine learning models were trained to distinguish between the two metabolic states.

**Figure 9:**
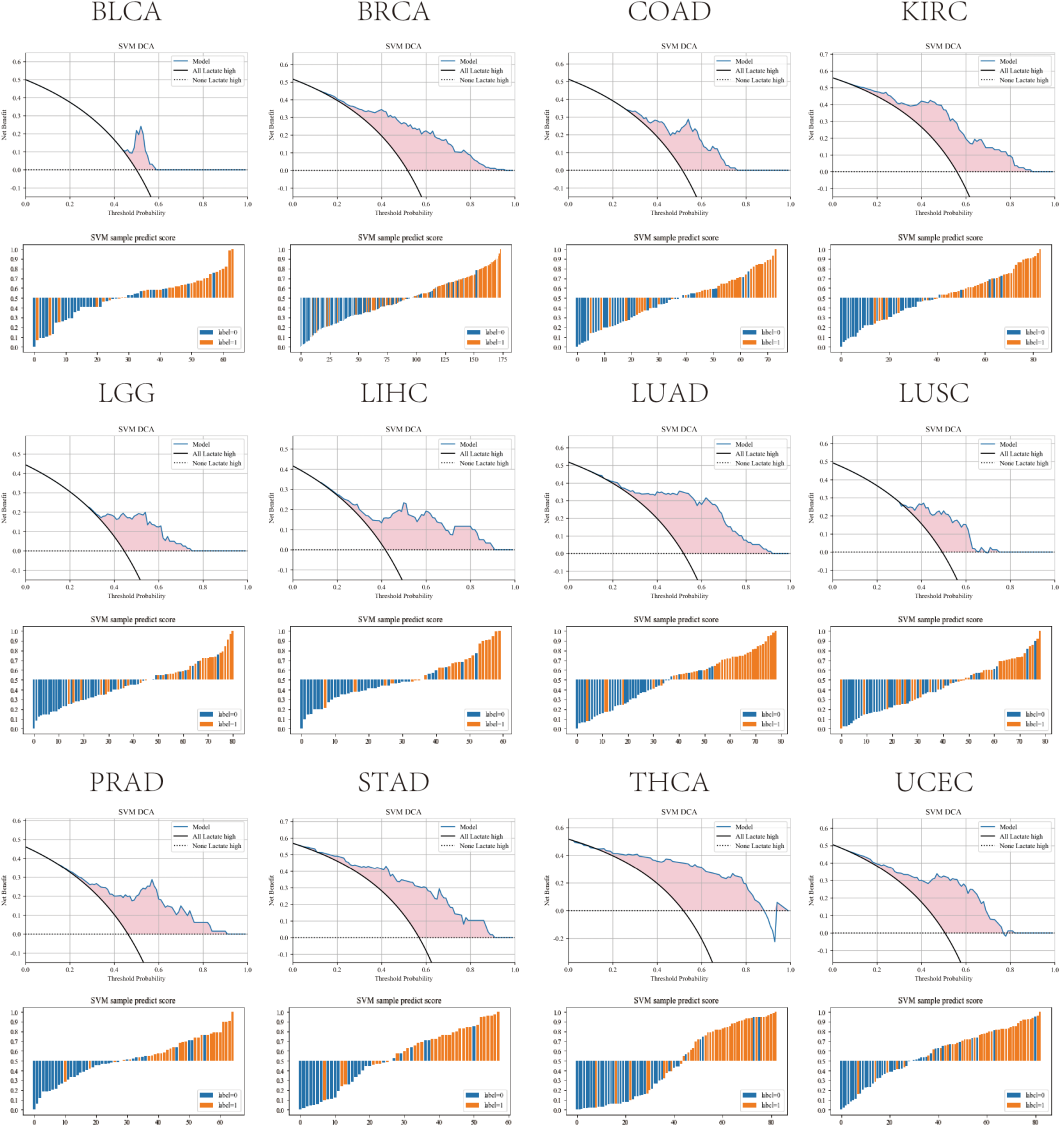
DCA and SVM prediction score distributions across multiple TCGA solid tumors (excluding HNSCC). DCA comparing the net clinical benefit of the SVM models, highlighting the potential clinical utility of each model across a range of threshold probabilities. Sample-level prediction score distributions for the SVM models, demonstrating clear separation between predicted LAC_H (label = 1) and LAC_L (label = 0) groups.

Among all tested algorithms, the SVM model consistently achieved the best cross-cancer performance, with AUROC values ranging from 0.78 to 0.89 across test cohorts (**Figure 8B**). The highest predictive accuracies were observed in KIRC, LUAD, LIHC, PRAD, STAD, THCA and UCEC with AUCs exceeding 0.85, while other tumor types such as BLCA, BRCA, COAD and LGG also demonstrated strong discriminative ability (AUC = 0.80-0.85). DCA further confirmed the superior clinical utility of the SVM model compared with other classifiers (**Supplementary Table 8**; **Figure 9**). Moreover, the probability distributions of predicted scores exhibited clear separation between LAC_H and LAC_L samples across all tumor types, supporting the model’s robustness and reproducibility. Detailed model construction and evaluation processes, including LASSO feature selection, ROC analysis, decision curve analysis, and sample-level prediction score distributions for each tumor type, are provided in **Supplementary Figures S5**-**S16**.

Collectively, these results demonstrate that the proposed pathology-based framework, particularly the SVM classifier, can accurately and generally infer lactate metabolic activity across diverse solid tumors, underscoring its robustness, scalability, and translational potential as a morphology-derived biomarker for tumor metabolic phenotyping.

## 4 Discussion

Lactate metabolism represents a central hallmark of tumor metabolic reprogramming and plays a critical role in shaping the immune and therapeutic landscape of malignancies. In this study, we systematically characterized a lactate-related gene set and demonstrated its biological significance in HNSCC. Through integrative multi-omic, single-cell, and histopathological analyses, we revealed that elevated lactate metabolic activity is associated with an immunosuppressive TME and enhanced resistance to radiotherapy. Furthermore, we established and externally validated a deep learning-based model that can accurately predict tumor lactate metabolic states directly from routine H&E stained WSIs, bridging metabolic profiling with practical clinical pathology. Importantly, validation in an independent real-world SAZHU-HNSCC cohort demonstrated concordance between model-predicted lactate states and protein-level expression of canonical lactate metabolism markers (LDHA and MCT1), supporting the translational robustness of this digital biomarker.

Notably, we further found that the lactate-related gene set was closely associated with immunotherapy response, with higher lactate scores correlating with reduced sensitivity to ICIs and poorer clinical outcomes (28, 35, 36). Mechanistically, hypoxia- and oncogene-driven glycolysis fuels monocarboxylate transporter (primarily MCT4/SLC16A3 and MCT1/SLC16A1)-mediated lactate efflux, which not only sustains tumor cell survival and redox balance under metabolic stress but also acidifies the TME. This acidification impairs immune-cell infiltration and effector function, skewing myeloid polarization, and dampening type I/II interferon signaling (9, 37). The extracellular acidosis and lactate accumulation collectively remodel the tumor-immune interface, reinforcing an immunosuppressive milieu that limits the efficacy of immune checkpoint blockade (7, 38). In HNSCC, Wang et al. reported elevated histone H3 lysine lactylation (such as H3K9la and H3K18la) which activates IL-11-JAK2-STAT3 signaling to drive CD8^+^ T-cell exhaustion, and correlates with poor responsiveness to ICIs (39). Together, these concordant molecular and morphological patterns validate the robustness of our image-based metabolic model and establish a clear mechanistic link between lactate-driven metabolic reprogramming and immune suppression in HNSCC. Notably, the deep learning model learned morphology-associated representations correlated with these biological states, suggesting that immune exclusion and compact tumor architecture may serve as visual surrogates of lactate-enriched metabolic programs.

Our results revealed that tumors with higher lactate-related gene set scores exhibited greater resistance to radiotherapy, reinforcing the concept that lactate metabolism plays a critical role in radioresistance. This observation is consistent with previous reports demonstrating that elevated intratumoral lactate levels are associated with poor radiation response (40, 41). As reported by Quennet et al., high lactate concentrations in HNSCC are significantly correlated with reduced overall survival and increased recurrence following adjuvant radiotherapy (42). These findings from prior studies further support the potential clinical relevance of lactate metabolism in treatment response. Mechanistically, enhanced lactate production enables tumor cells to preserve redox homeostasis and attenuate radiation-induced oxidative stress by scavenging reactive oxygen species. Upregulation of LDHA-the key enzyme catalyzing pyruvate-to-lactate conversion-amplifies this protective effect, whereas LDHA inhibition increases radiosensitivity by promoting apoptosis and impairing DNA repair (43). Extending these findings, Liu et al. identified TAB182 as an upstream regulator of this process, showing that TAB182 activates SP1/c-MYC-dependent LDHA transcription to drive lactate production, metabolic reprogramming, and antioxidant defense, ultimately fostering radiation resistance (44). Consistent with these observations, Ito et al. demonstrated that the glycolysis inhibitor oxamate enhances radiosensitivity in glioblastoma cells by delaying DNA repair, inducing apoptosis and senescence, and suppressing cancer stem cell properties and EMT-related pathways (45). Collectively, these findings indicate that activation of the glycolysis–lactate axis reinforces cellular resilience to radiation-induced damage. Targeting this metabolic pathway may thus represent a promising therapeutic approach to enhance radiosensitivity and overcome lactate-driven radioresistance.

Moreover, our results illustrate how deep learning-based histopathological analysis extends the translational relevance of lactate metabolism in cancer. By capturing subtle morphological cues, such as reduced immune infiltration and compact tumor cell nests, our model bridges metabolic biology with diagnostic pathology, providing a visual readout of tumor metabolic states. These image-derived features are consistent with the biological hallmarks of lactate-enriched tumors and underscore the potential of digital pathology as a surrogate biomarker for metabolic phenotyping. Beyond mechanistic understanding, such non-invasive computational tools may enable real-time patient stratification, particularly in settings where molecular profiling is limited.

Finally, from a translational perspective, the digital lactate score may complement established biomarkers such as PD-L1 immunohistochemistry and tumor mutational burden (TMB), as it reflects metabolic-immune interactions within the tumor microenvironment rather than immune activation alone. This biomarker could potentially help explain heterogeneous responses to immune checkpoint inhibitors and support patient stratification. In addition, given the association between lactate-high tumors and immunosuppressive signaling, the score may provide a rationale for exploring combination strategies targeting the lactate axis (e.g., LDHA or MCT1 inhibition) together with immune checkpoint blockade in future prospective studies.

Several limitations of this study should be acknowledged. First, although the pathology-based model demonstrated robust performance and external validation, the SAZHU-HNSCC cohort size for IHC validation was modest and derived from a single center. The integrated lactate prediction score was based on retrospective modeling, and prospective validation is warranted to assess its clinical utility. In addition, while the model captures morphology-associated metabolic states, it does not directly quantify intratumoral lactate concentrations, which is partly constrained by tissue availability, as reliable lactate measurements generally require fresh tumor specimens rather than archived FFPE samples. Furthermore, despite the visualization of attention maps, the overall framework retains characteristics of a high-dimensional, representation-driven model. The transformer module serves to generate slide-level embeddings, and final classification is performed using LASSO-selected PCA features and downstream machine learning classifiers. As a result, the predictive features are abstract and do not permit direct, structure-level mapping to specific histomorphological elements such as defined cellular arrangements, stromal architecture, or necrosis patterns. Although qualitative morphological differences were observed between groups, the current approach lacks fully intuitive, mechanism-level interpretability for pathologists and should therefore be considered partially “black-box” in nature. Future studies integrating spatial metabolomics and prospective clinical trials will be essential to further refine and validate this digital biomarker.

Together, these concordant molecular and morphological patterns support the biological relevance of our image-based metabolic model and suggest a potential link between lactate-driven metabolic reprogramming and immune suppression in HNSCC. Importantly, our deep learning-based pathology model provides a novel, non-invasive approach to infer lactate metabolic states directly from H&E-stained WSIs, which may complement genomic and transcriptomic profiling in clinical practice.

## Data Availability

Due to ethical constraints and patient privacy protection, FFPE tissue specimens and clinical data from the SHAZU-HNSCC cohort cannot be made publicly available. De-identified data may be shared with qualified researchers for academic purposes only, upon reasonable request to the corresponding authors and approval by the local ethics committee. Other datasets used in this study are publicly available from their respective sources. The names of the repository/repositories and accession number(s) can be found in the article/**Supplementary Material**.

## Supplementary material

Supplementary Figure 1 Prognostic value of the lactate-related gene set score in HNSCC patients receiving adjuvant radiotherapy. The upper forest plots present hazard ratios (HRs) and 95% confidence intervals (CIs) derived from univariate Cox proportional hazards regression analyses for overall survival (OS) **(A)**, progression-free interval (PFI) **(B)**, and disease-specific survival (DSS) **(C)**. The lower forest plots display HRs and 95% CIs from multivariate Cox proportional hazards regression analyses adjusted for the indicated clinicopathological variables, with OS **(A)**, PFI **(B)**, and DSS **(C)** as clinical endpoints. For categorical variables, the group listed to the left of the “Feature” column is defined as the reference category.

Supplementary Figure 2 Single-cell inferCNV analysis and spatial localization of lactate-related genes in HNSCC. **(A)** InferCNV analysis of epithelial and CD4^+^ T cells from integrated single-cell RNA-seq datasets. Large-scale chromosomal copy number variations (CNVs) were visualized to distinguish malignant epithelial cells (red signal indicates chromosomal gains, blue indicates losses). **(B)** UMAP visualization of 38 lactate-related genes broadly expressed across multiple cell types within the HNSCC microenvironment. **(C–E)** UMAP plots showing cell type-specific lactate-related gene expression, including SLC2A2 (mast cells), NARS2 (pDCs), and HCAR1 (tumor-associated neutrophils, TANs).

Supplementary Figure 3 Dot plots showing differences in ICI-related LR pairs between LAC_H and LAC_L groups. Each dot represents a cancer type, with red dots indicating higher levels in LAC_H and blue dots indicating higher levels in LAC_L. The panels display four categories of LR pairs: **(A)** immune checkpoint pairs, **(B)** costimulatory pairs, **(C)** MHC-I–related pairs, and **(D)** the IFNG–IFNGR1 pair.

Supplementary Figure 4 Dot plots showing differences in immune specific functional states and TLS-related immune signatures between LAC_H and LAC_L groups. Each dot represents a cancer type, with red dots indicating higher signature levels in LAC_H and blue dots indicating higher levels in LAC_L. The panels include T cell exhaustion (A), macrophage polarization (B) and multiple TLS-associated features (C): tumor-infiltrating lymphocytes (TILs), plasma cell infiltration, tertiary lymphoid structures (TLS), and high-endothelial venule-associated lymphoid aggregates (HALA.TLS). Delta values represent LAC_H − LAC_L.

Supplementary Figure 5 Prediction of LAC_H and LAC_L groups based on pathological machine learning models in BLCA. **(A–C)** A total of 512-dimensional pathology-derived features were extracted from the transformer-based WSI model and reduced to 128 dimensions using PCA. LASSO regression identified 9 key features for downstream modeling. **(D)** ROC curves and corresponding AUC values of XGBoost, GradientBoosting and LightGBM for LAC_H/LAC_L classification, evaluated on training and test cohorts. **(E)** DCA comparing the net clinical benefit of the three models, illustrating the potential utility of each model across varying threshold probabilities. **(F)** Sample-level prediction score distributions for XGBoost, GradientBoosting and LightGBM models, showing separation between predicted LAC_H (label = 1) and LAC_L (label = 0) groups.

Supplementary Figure 6 Prediction of LAC_H and LAC_L groups based on pathological machine learning models in BRCA. **(A–C)** A total of 512-dimensional pathology-derived features were extracted from the transformer-based WSI model and reduced to 128 dimensions using PCA. LASSO regression identified 64 key features for downstream modeling. **(D)** ROC curves and corresponding AUC values of XGBoost, GradientBoosting and LightGBM for LAC_H/LAC_L classification, evaluated on training and test cohorts. **(E)** DCA comparing the net clinical benefit of the three models, illustrating the potential utility of each model across varying threshold probabilities. **(F)** Sample-level prediction score distributions for XGBoost, GradientBoosting and LightGBM models, showing separation between predicted LAC_H (label = 1) and LAC_L (label = 0) groups.

Supplementary Figure 7 Prediction of LAC_H and LAC_L groups based on pathological machine learning models in COAD. **(A–C)** A total of 512-dimensional pathology-derived features were extracted from the transformer-based WSI model and reduced to 128 dimensions using PCA. LASSO regression identified 5 key features for downstream modeling. **(D)** ROC curves and corresponding AUC values of XGBoost, GradientBoosting and LightGBM for LAC_H/LAC_L classification, evaluated on training and test cohorts. **(E)** DCA comparing the net clinical benefit of the three models, illustrating the potential utility of each model across varying threshold probabilities. **(F)** Sample-level prediction score distributions for XGBoost, GradientBoosting and LightGBM models, showing separation between predicted LAC_H (label = 1) and LAC_L (label = 0) groups.

Supplementary Figure 8 Prediction of LAC_H and LAC_L groups based on pathological machine learning models in KIRC. **(A–C)** A total of 512-dimensional pathology-derived features were extracted from the transformer-based WSI model and reduced to 128 dimensions using PCA. LASSO regression identified 65 key features for downstream modeling. **(D)** ROC curves and corresponding AUC values of XGBoost, GradientBoosting and LightGBM for LAC_H/LAC_L classification, evaluated on training and test cohorts. **(E)** DCA comparing the net clinical benefit of the three models, illustrating the potential utility of each model across varying threshold probabilities. **(F)** Sample-level prediction score distributions for XGBoost, GradientBoosting and LightGBM models, showing separation between predicted LAC_H (label = 1) and LAC_L (label = 0) groups.

Supplementary Figure 9 Prediction of LAC_H and LAC_L groups based on pathological machine learning models in LGG. **(A–C)** A total of 512-dimensional pathology-derived features were extracted from the transformer-based WSI model and reduced to 128 dimensions using PCA. LASSO regression identified 24 key features for downstream modeling. **(D)** ROC curves and corresponding AUC values of XGBoost, GradientBoosting and LightGBM for LAC_H/LAC_L classification, evaluated on training and test cohorts. **(E)** DCA comparing the net clinical benefit of the three models, illustrating the potential utility of each model across varying threshold probabilities. **(F)** Sample-level prediction score distributions for XGBoost, GradientBoosting and LightGBM models, showing separation between predicted LAC_H (label = 1) and LAC_L (label = 0) groups.

Supplementary Figure 10 Prediction of LAC_H and LAC_L groups based on pathological machine learning models in LIHC. **(A–C)** A total of 512-dimensional pathology-derived features were extracted from the transformer-based WSI model and reduced to 128 dimensions using PCA. LASSO regression identified 30 key features for downstream modeling. **(D)** ROC curves and corresponding AUC values of XGBoost, GradientBoosting and LightGBM for LAC_H/LAC_L classification, evaluated on training and test cohorts. **(E)** DCA comparing the net clinical benefit of the three models, illustrating the potential utility of each model across varying threshold probabilities. **(F)** Sample-level prediction score distributions for XGBoost, GradientBoosting and LightGBM models, showing separation between predicted LAC_H (label = 1) and LAC_L (label = 0) groups.

Supplementary Figure 11 Prediction of LAC_H and LAC_L groups based on pathological machine learning models in LUAD. **(A–C)** A total of 512-dimensional pathology-derived features were extracted from the transformer-based WSI model and reduced to 128 dimensions using PCA. LASSO regression identified 26 key features for downstream modeling. **(D)** ROC curves and corresponding AUC values of XGBoost, GradientBoosting and LightGBM for LAC_H/LAC_L classification, evaluated on training and test cohorts. **(E)** DCA comparing the net clinical benefit of the three models, illustrating the potential utility of each model across varying threshold probabilities. **(F)** Sample-level prediction score distributions for XGBoost, GradientBoosting and LightGBM models, showing separation between predicted LAC_H (label = 1) and LAC_L (label = 0) groups.

Supplementary Figure 12 Prediction of LAC_H and LAC_L groups based on pathological machine learning models in LUSC. **(A–C)** A total of 512-dimensional pathology-derived features were extracted from the transformer-based WSI model and reduced to 128 dimensions using PCA. LASSO regression identified 5 key features for downstream modeling. **(D)** ROC curves and corresponding AUC values of XGBoost, GradientBoosting and LightGBM for LAC_H/LAC_L classification, evaluated on training and test cohorts. **(E)** DCA comparing the net clinical benefit of the three models, illustrating the potential utility of each model across varying threshold probabilities. **(F)** Sample-level prediction score distributions for XGBoost, GradientBoosting and LightGBM models, showing separation between predicted LAC_H (label = 1) and LAC_L (label = 0) groups.

Supplementary Figure 13 Prediction of LAC_H and LAC_L groups based on pathological machine learning models in PRAD. **(A–C)** A total of 512-dimensional pathology-derived features were extracted from the transformer-based WSI model and reduced to 128 dimensions using PCA. LASSO regression identified 53 key features for downstream modeling. **(D)** ROC curves and corresponding AUC values of XGBoost, GradientBoosting and LightGBM for LAC_H/LAC_L classification, evaluated on training and test cohorts. **(E)** DCA comparing the net clinical benefit of the three models, illustrating the potential utility of each model across varying threshold probabilities. **(F)** Sample-level prediction score distributions for XGBoost, GradientBoosting and LightGBM models, showing separation between predicted LAC_H (label = 1) and LAC_L (label = 0) groups.

Supplementary Figure 14 Prediction of LAC_H and LAC_L groups based on pathological machine learning models in STAD. **(A–C)** A total of 512-dimensional pathology-derived features were extracted from the transformer-based WSI model and reduced to 128 dimensions using PCA. LASSO regression identified 36 key features for downstream modeling. **(D)** ROC curves and corresponding AUC values of XGBoost, GradientBoosting and LightGBM for LAC_H/LAC_L classification, evaluated on training and test cohorts. **(E)** DCA comparing the net clinical benefit of the three models, illustrating the potential utility of each model across varying threshold probabilities. **(F)** Sample-level prediction score distributions for XGBoost, GradientBoosting and LightGBM models, showing separation between predicted LAC_H (label = 1) and LAC_L (label = 0) groups.

Supplementary Figure 15 Prediction of LAC_H and LAC_L groups based on pathological machine learning models in THCA. **(A–C)** A total of 512-dimensional pathology-derived features were extracted from the transformer-based WSI model and reduced to 128 dimensions using PCA. LASSO regression identified 47 key features for downstream modeling. **(D)** ROC curves and corresponding AUC values of XGBoost, GradientBoosting and LightGBM for LAC_H/LAC_L classification, evaluated on training and test cohorts. **(E)** DCA comparing the net clinical benefit of the three models, illustrating the potential utility of each model across varying threshold probabilities. **(F)** Sample-level prediction score distributions for XGBoost, GradientBoosting and LightGBM models, showing separation between predicted LAC_H (label = 1) and LAC_L (label = 0) groups.

Supplementary Figure 16 Prediction of LAC_H and LAC_L groups based on pathological machine learning models in UCEC. **(A–C)** A total of 512-dimensional pathology-derived features were extracted from the transformer-based WSI model and reduced to 128 dimensions using PCA. LASSO regression identified 56 key features for downstream modeling. **(D)** ROC curves and corresponding AUC values of XGBoost, GradientBoosting and LightGBM for LAC_H/LAC_L classification, evaluated on training and test cohorts. **(E)** DCA comparing the net clinical benefit of the three models, illustrating the potential utility of each model across varying threshold probabilities. **(F)** Sample-level prediction score distributions for XGBoost, GradientBoosting and LightGBM models, showing separation between predicted LAC_H (label = 1) and LAC_L (label = 0) groups.
