# Supplementary material for: Tumor lactate metabolism shapes immune suppression and therapeutic resistance revealed by integrative multi-omics and digital pathology
Source: Front Immunol. 2026 Mar 26;17:1797798. doi: 10.3389/fimmu.2026.1797798 (PMC13062246; doi:10.3389/fimmu.2026.1797798)
Supplement: Supplementary Figure 1 — Prognostic value of the lactate-related gene set score in HNSCC patients receiving adjuvant radiotherapy. The upper forest plots present hazard ratios (HRs) and 95% confidence intervals (CIs) derived from univariate Cox proportional hazards regression analyses for overall survival (OS) (A), progression-free interval (PFI) (B), and disease-specific survival (DSS) (C). The lower forest plots display HRs and 95% CIs from multivariate Cox proportional hazards regression analyses adjusted for the indicated clinicopathological variables, with OS (A), PFI (B), and DSS (C) as clinical endpoints. For categorical variables, the group listed to the left of the “Feature” column is defined as the reference category. [file DataSheet1.docx]

**Single-cell Transcriptomic Integration**

In September 2025, three HNSCC single-cell RNA sequencing datasets (GSE181919(1), GSE182227(2), and GSE234933(3)) were acquired from the NCBI GEO, and in August 2025, an additional dataset (GSE290927(4)) was obtained. All four datasets were processed into Seurat objects for downstream analysis using the Seurat v4.4.0 R package (5).

To ensure data quality, we applied stringent filtering criteria to the single-cell RNA-seq datasets. For GSE181919(1), GSE182227(2) and GSE290927(4), cells were retained if they met all of the following conditions: possessing between 250 and 5,000 detected features, exhibiting less than 10% mitochondrial gene expression, less than 1% hemoglobin gene expression, more than 10% ribosomal gene expression, and containing over 1,000 total RNA counts. For the GSE234933(3) dataset, the same criteria were enforced, except the cut-off for the detected features at which cells were excluded, which was set to > 2,500.

We normalized and performed feature selection on the single-cell RNA-seq data using the NormalizeData function, restricting variable features to the top 5,000 genes. Following feature identification, we refined the gene list by excluding specific gene sets, including stress-related genes, long intergenic non-coding RNAs (LINC), mitochondrial genes, and immunoglobulin genes. Additionally, we removed undesired gene categories by filtering out genes with prefixes such as "MT-", "RP[SL]", and "TR[ABDG]", as well as those with suffixes like "-AS" and "-DT". Genes representing pseudogenes or certain immunoglobulin variants (e.g., IGHV, IGLV) were also excluded to ensure the accuracy and relevance of the feature set (6).

After refining the feature list, principal component analysis (PCA) was performed using Seurat’s RunPCA function. To reduce dimensionality and facilitate clustering of the single-cell RNA-seq data, the optimal number of principal components (PCs) was determined. Across the entire cell population, the top 20 PCs were selected, accounting for more than 80% of the total variance. To correct for batch effects, the Harmony algorithm (7), was applied, followed by Uniform Manifold Approximation and Projection (UMAP) for visualization. Cell neighborhoods and clusters were then identified using Seurat’s FindNeighbors and FindClusters functions at a resolution of 0.8. Finally, cell clusters were annotated based on known biological cell types using canonical marker genes.

Furthermore, we employed the inferCNV package to detect copy number variations (CNVs) and differentiate malignant cells. Subsequently, cells were clustered using k-means clustering, and epithelial cells exhibiting abnormal CNV profiles were identified as potential malignant cells. Single-cell gene set enrichment scores were calculated using the AddModuleScore function, enabling the assessment of gene activity at the individual cell level.

**Integrating scRNA-seq and Bulk RNA-seq Data with BayesPrism**

ScRNA-seq data were integrated with bulk RNA-seq data, followed by correlation and outlier analyses using the BayesPrism package (8). After filtering and preprocessing the single-cell data, protein-coding genes were selected, and differential gene expression analysis was conducted. Finally, BayesPrism was employed to deconvolute the bulk RNA-seq data, enabling the estimation of cell type proportions within the bulk samples.

**Reference**

1. Choi JH, Lee BS, Jang JY, Lee YS, Kim HJ, Roh J, et al. Single-cell transcriptome profiling of the stepwise progression of head and neck cancer. Nat Commun. 2023;14(1):1055.

2. Puram SV, Mints M, Pal A, Qi Z, Reeb A, Gelev K, et al. Cellular states are coupled to genomic and viral heterogeneity in HPV-related oropharyngeal carcinoma. Nat Genet. 2023;55(4):640-50.

3. Bill R, Wirapati P, Messemaker M, Roh W, Zitti B, Duval F, et al. CXCL9:SPP1 macrophage polarity identifies a network of cellular programs that control human cancers. Science. 2023;381(6657):515-24.

4. Fu ZM, Bao YY, Dai LB, Zhong JT, Chen HC, Chen Z, et al. Comprehensive Single-Cell RNA Atlas of Human Laryngeal Normal, Preneoplastic, and Tumorigenic States. Clin Cancer Res. 2025;31(15):3332-43.

5. Hao Y, Hao S, Andersen-Nissen E, Mauck WM, 3rd, Zheng S, Butler A, et al. Integrated analysis of multimodal single-cell data. Cell. 2021;184(13):3573-87 e29.

6. Caushi JX, Zhang J, Ji Z, Vaghasia A, Zhang B, Hsiue EH, et al. Transcriptional programs of neoantigen-specific TIL in anti-PD-1-treated lung cancers. Nature. 2021;596(7870):126-32.

7. Korsunsky I, Millard N, Fan J, Slowikowski K, Zhang F, Wei K, et al. Fast, sensitive and accurate integration of single-cell data with Harmony. Nat Methods. 2019;16(12):1289-96.

8. Chu T, Wang Z, Pe'er D, Danko CG. Cell type and gene expression deconvolution with BayesPrism enables Bayesian integrative analysis across bulk and single-cell RNA sequencing in oncology. Nat Cancer. 2022;3(4):505-17.
