# Supplementary material for: Tumor lactate metabolism shapes immune suppression and therapeutic resistance revealed by integrative multi-omics and digital pathology
Source: Front Immunol. 2026 Mar 26;17:1797798. doi: 10.3389/fimmu.2026.1797798 (PMC13062246; doi:10.3389/fimmu.2026.1797798)
Supplement: Supplementary file 3 [file Presentation1.pptx]

## Slide 1
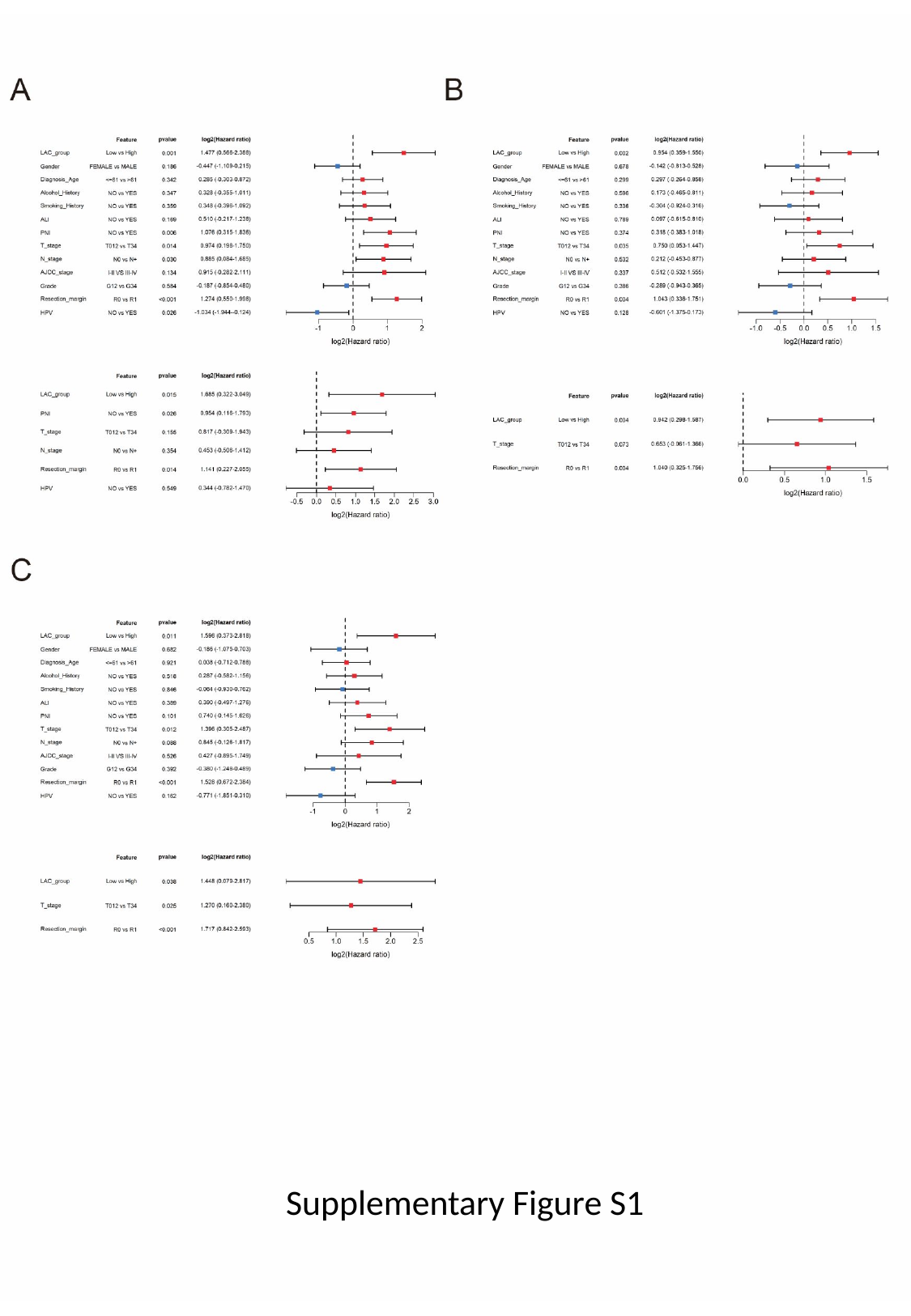

Supplementary Figure S1

## Slide 2
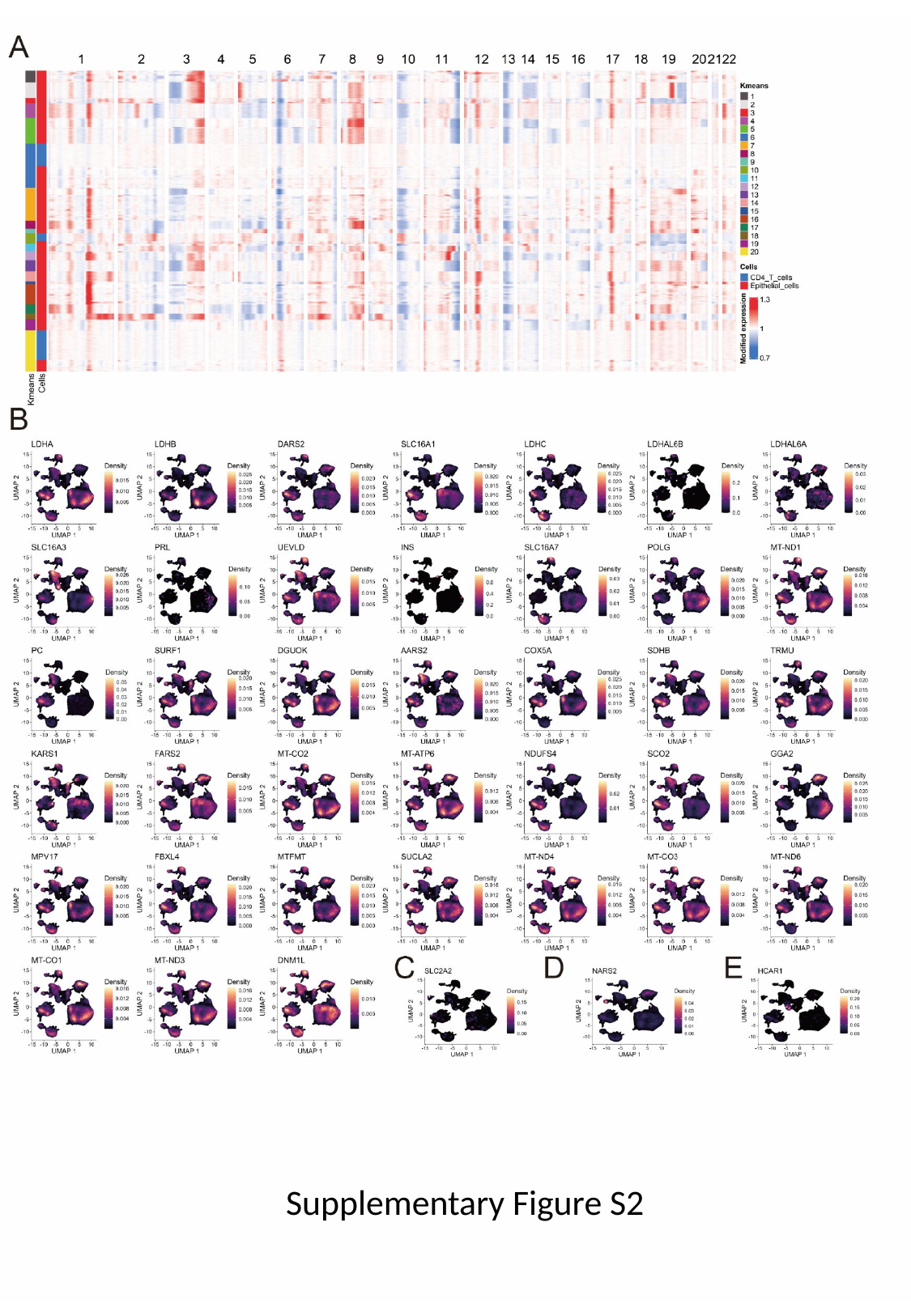

Supplementary Figure S2

## Slide 3
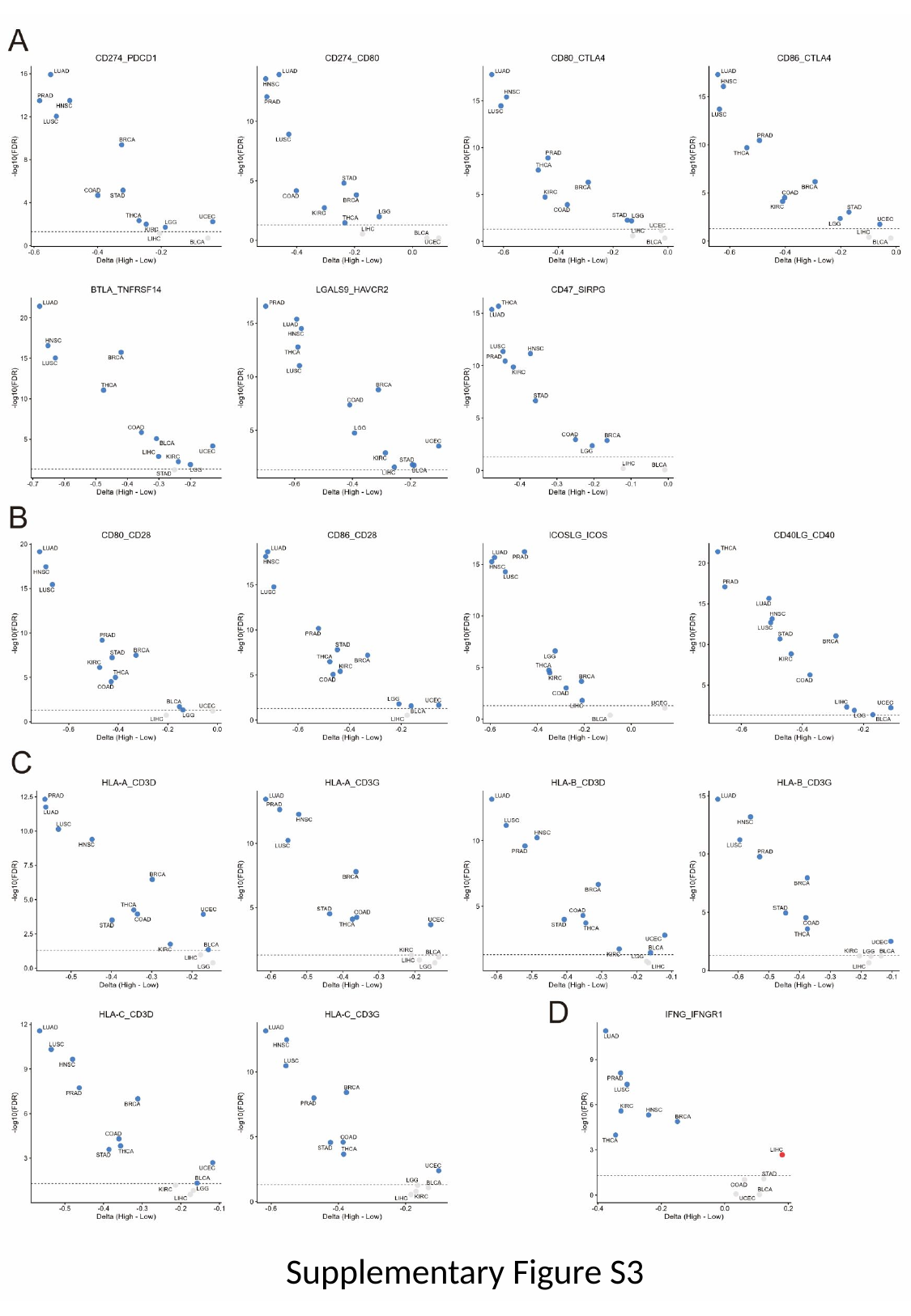

Supplementary Figure S3

## Slide 4
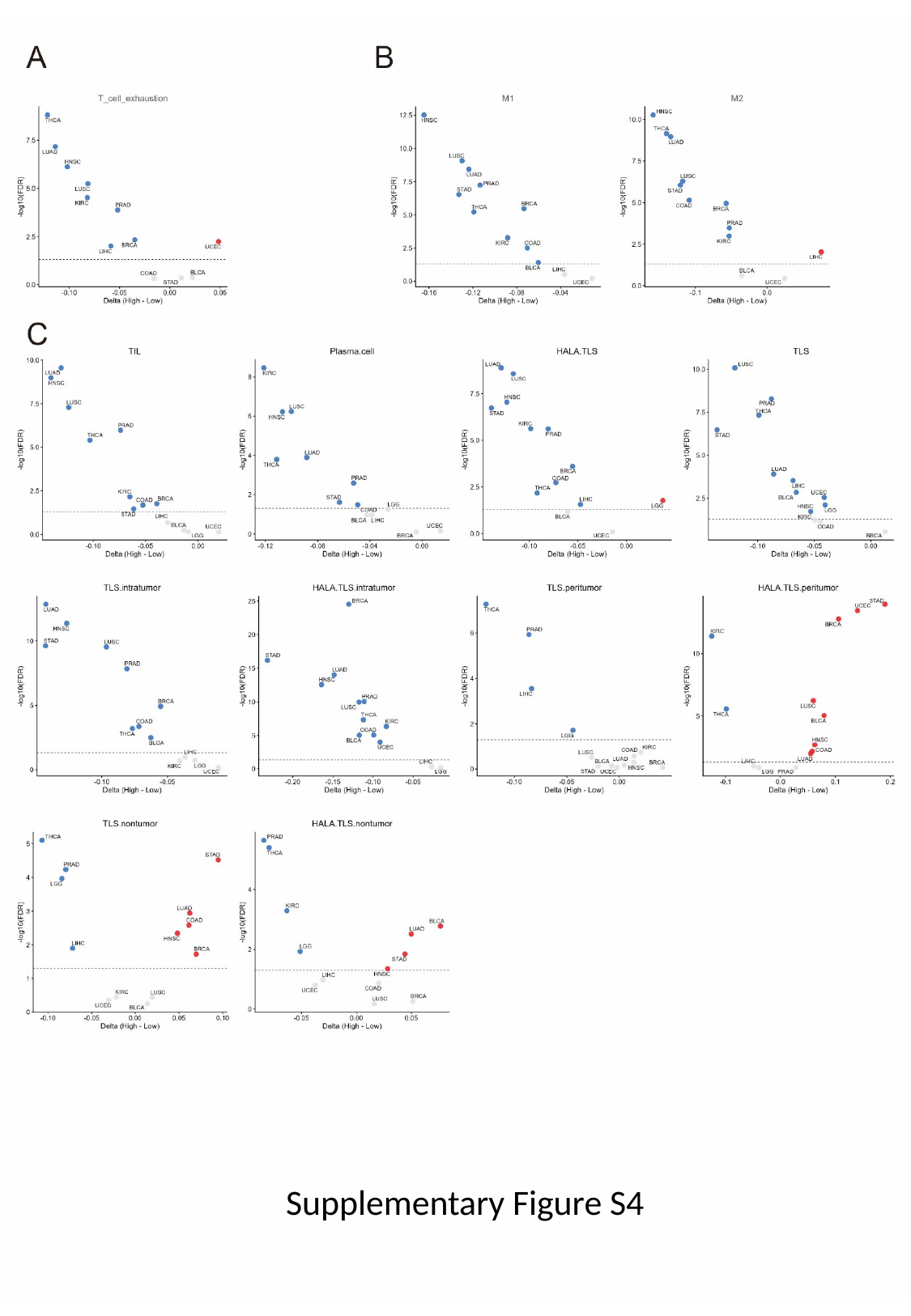

Supplementary Figure S4

## Slide 5
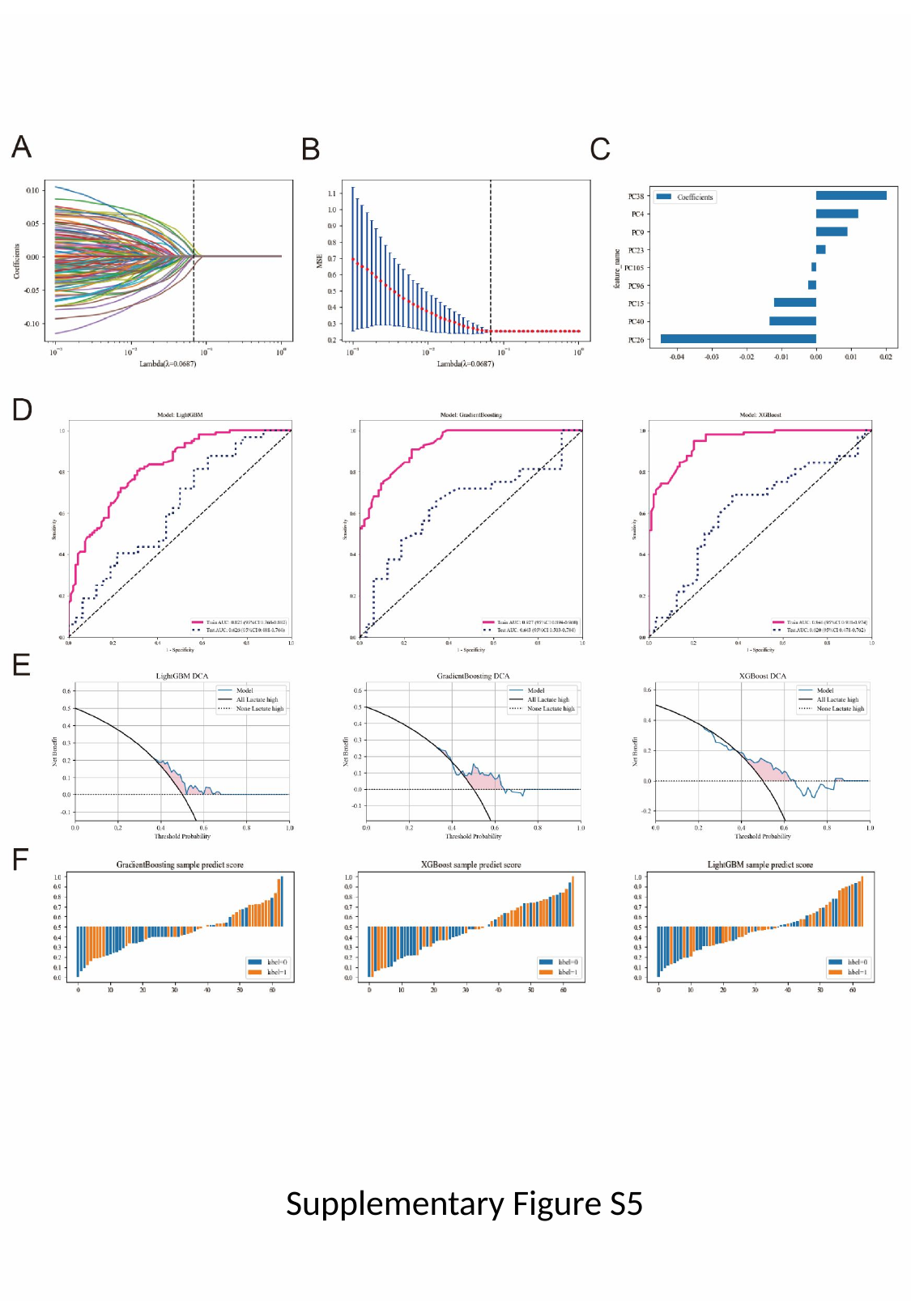

Supplementary Figure S5

## Slide 6
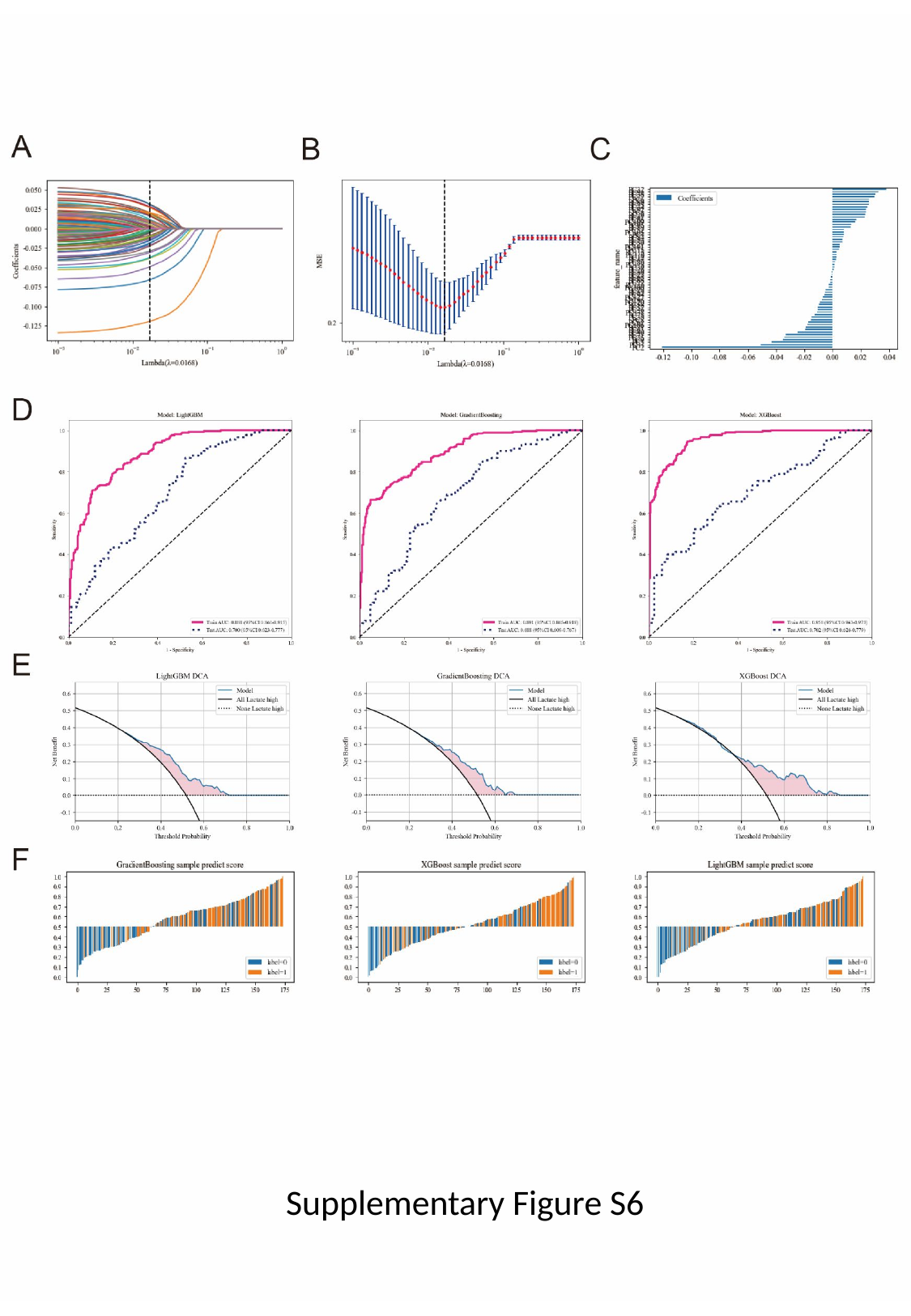

Supplementary Figure S6

## Slide 7
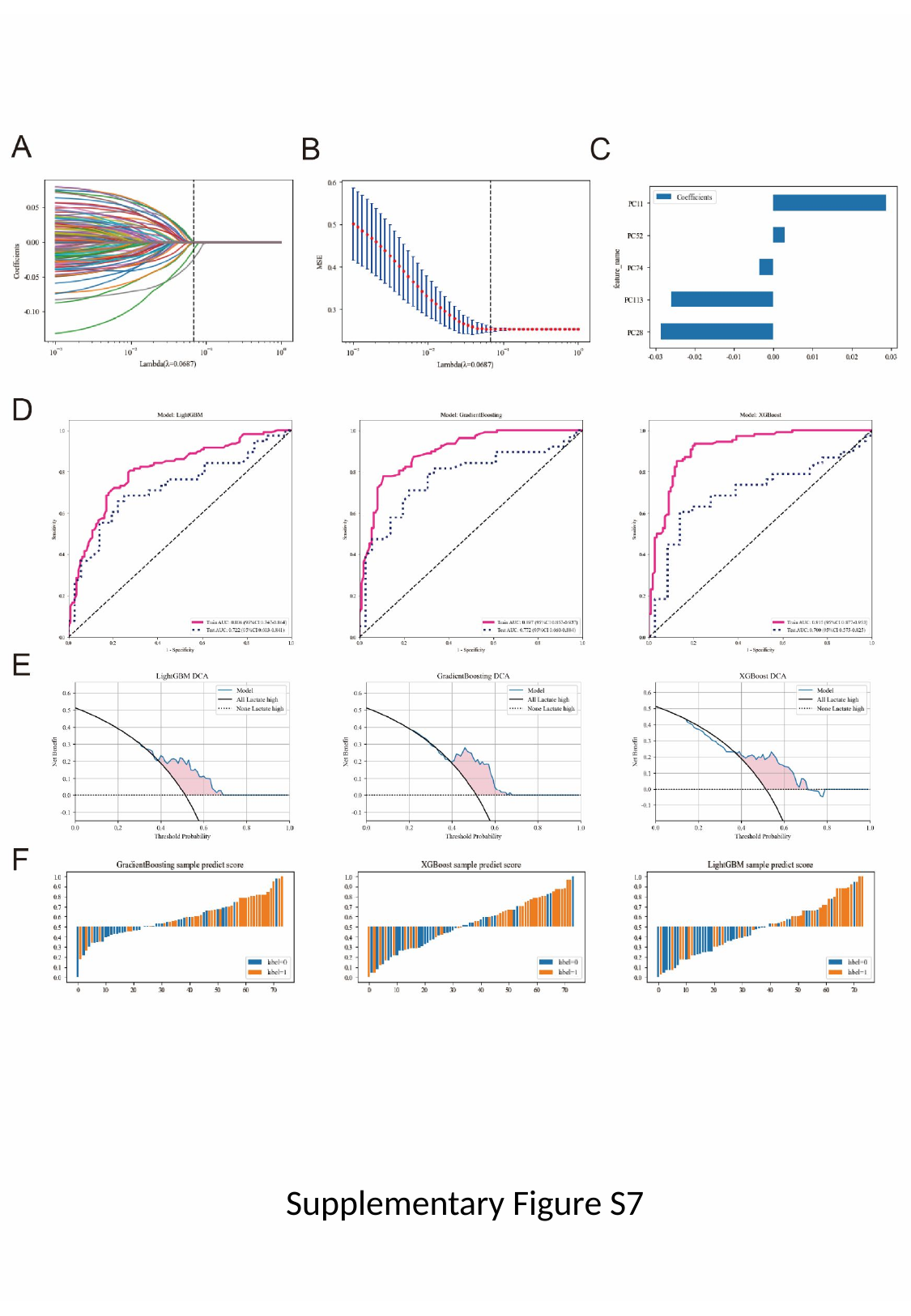

Supplementary Figure S7

## Slide 8
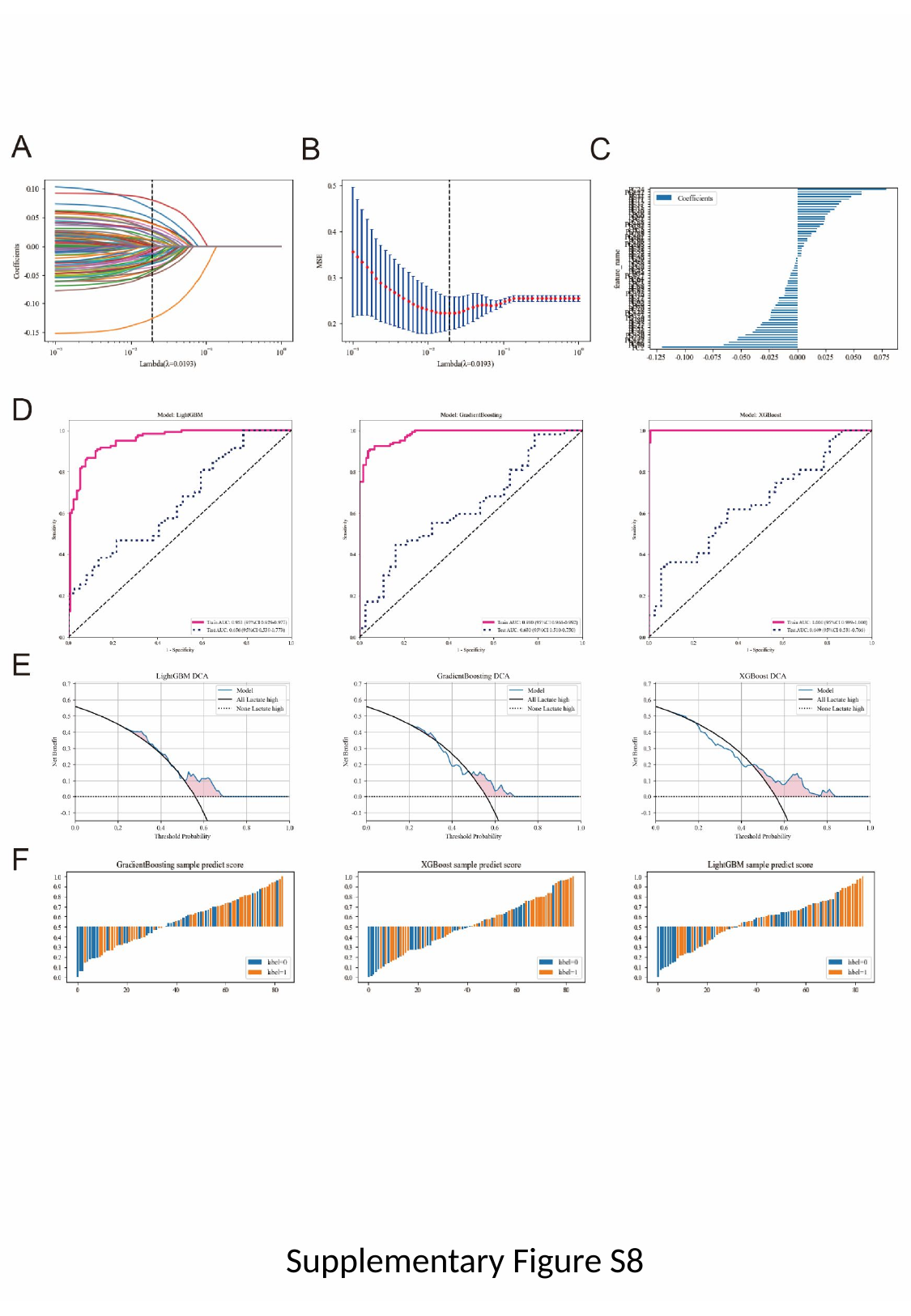

Supplementary Figure S8

## Slide 9
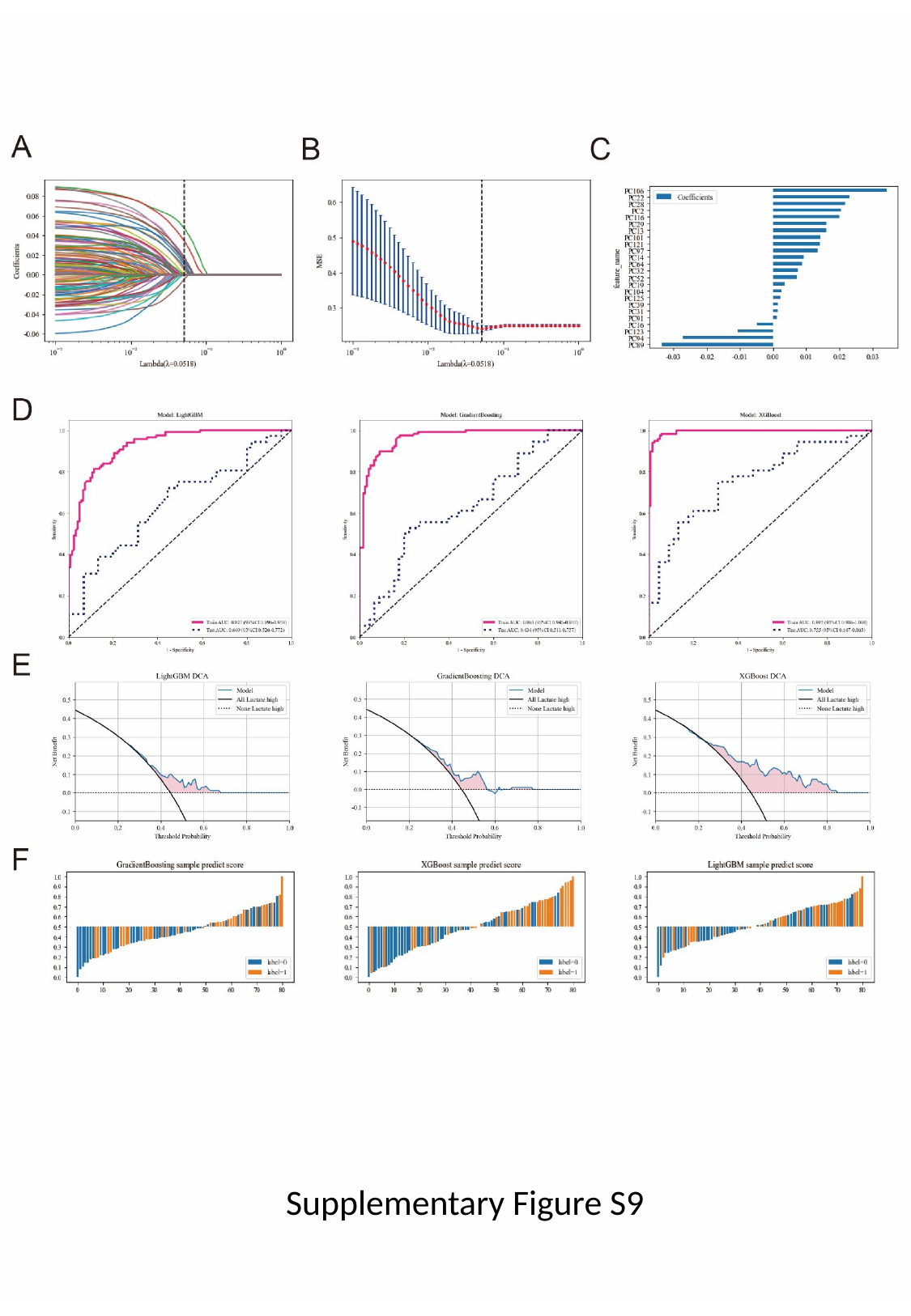

Supplementary Figure S9

## Slide 10
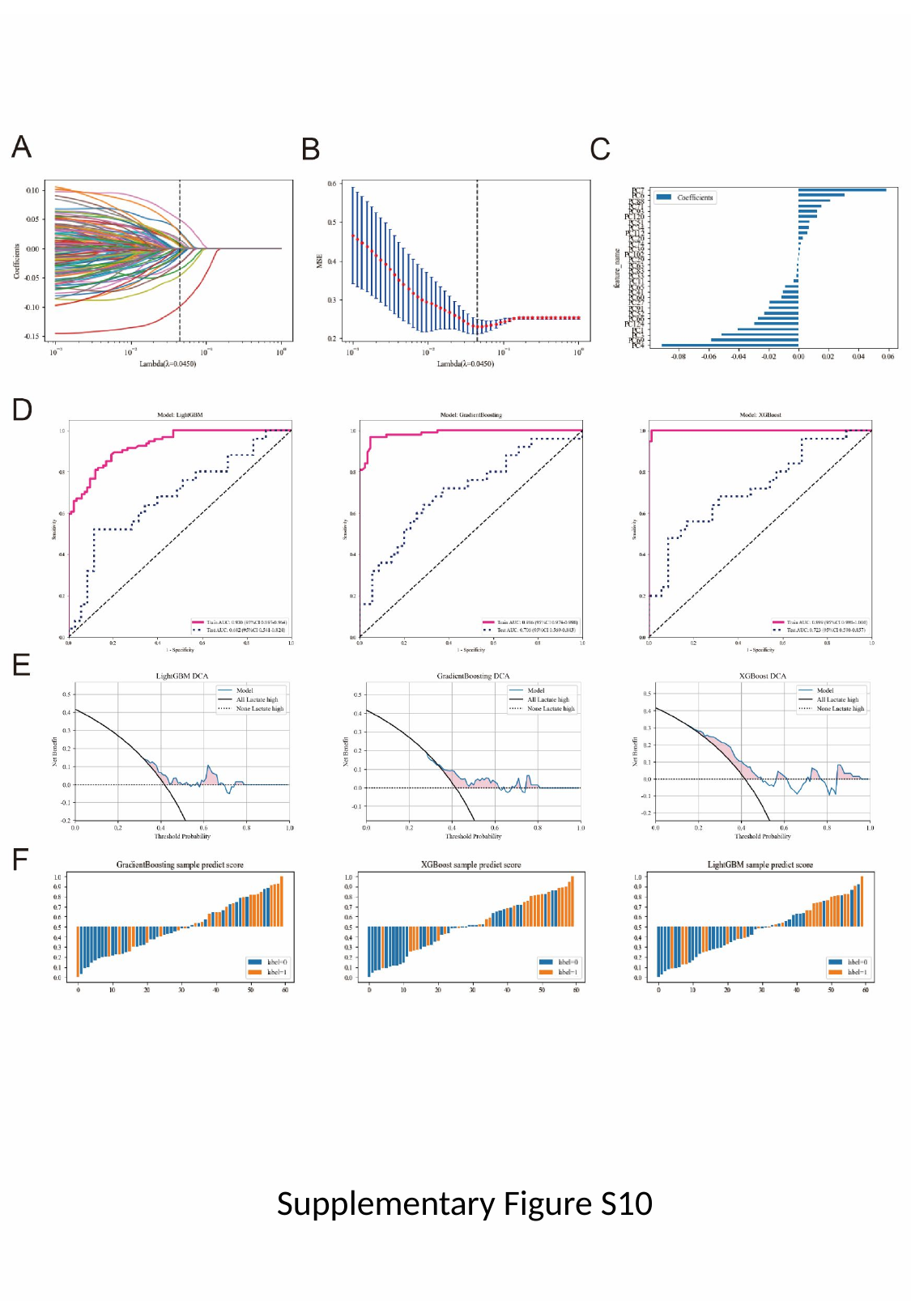

Supplementary Figure S10

## Slide 11
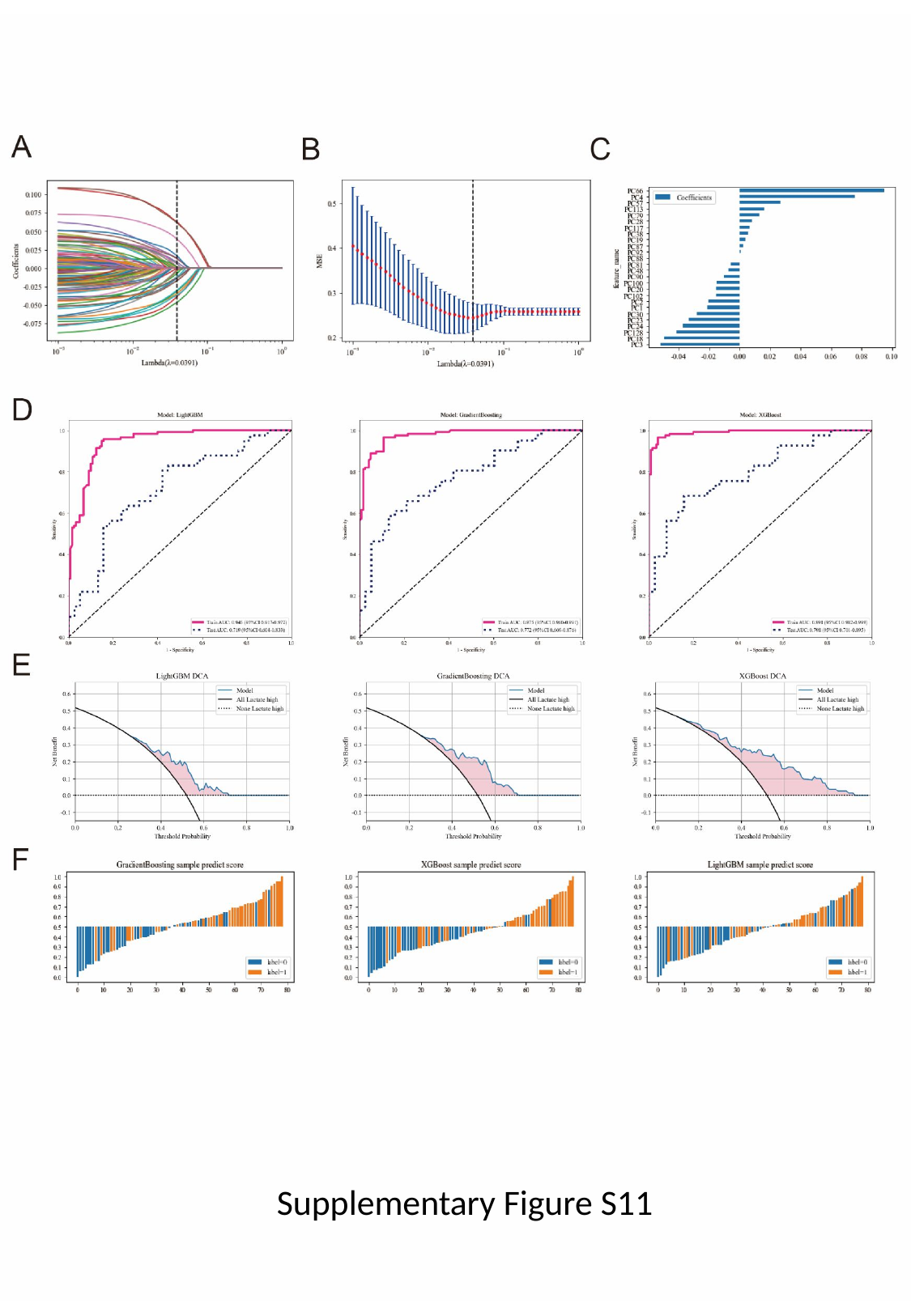

Supplementary Figure S11

## Slide 12
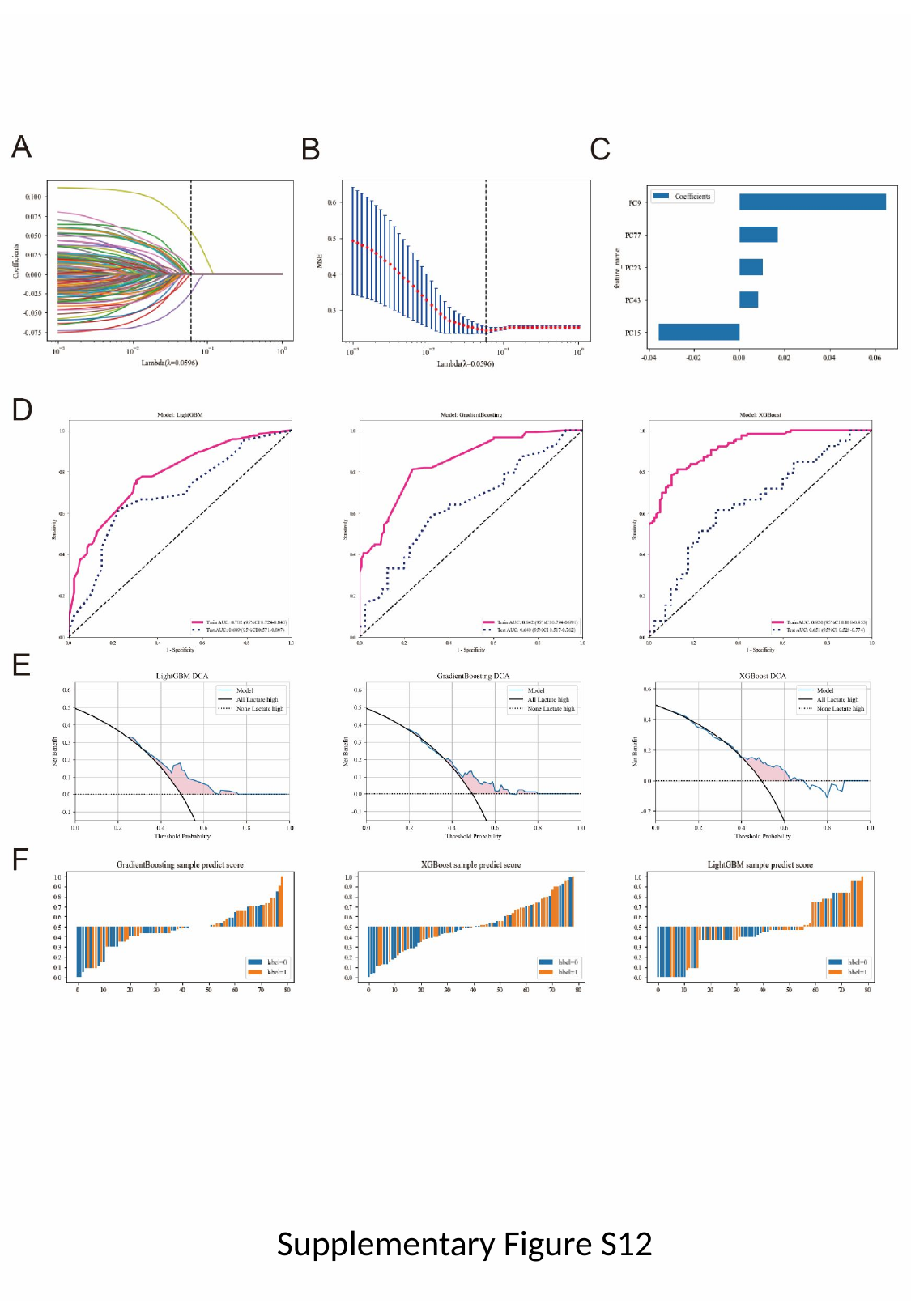

Supplementary Figure S12

## Slide 13
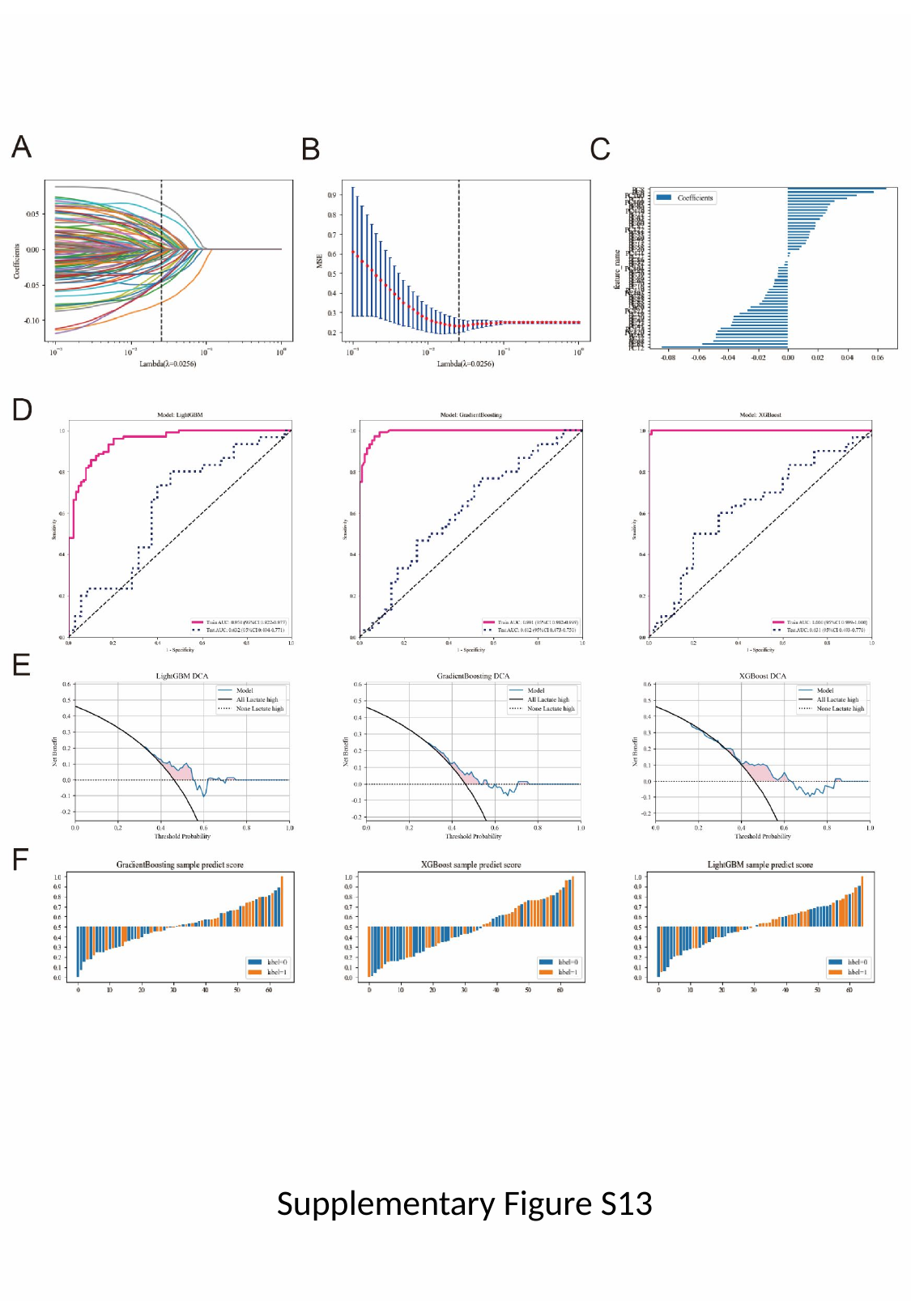

Supplementary Figure S13

## Slide 14
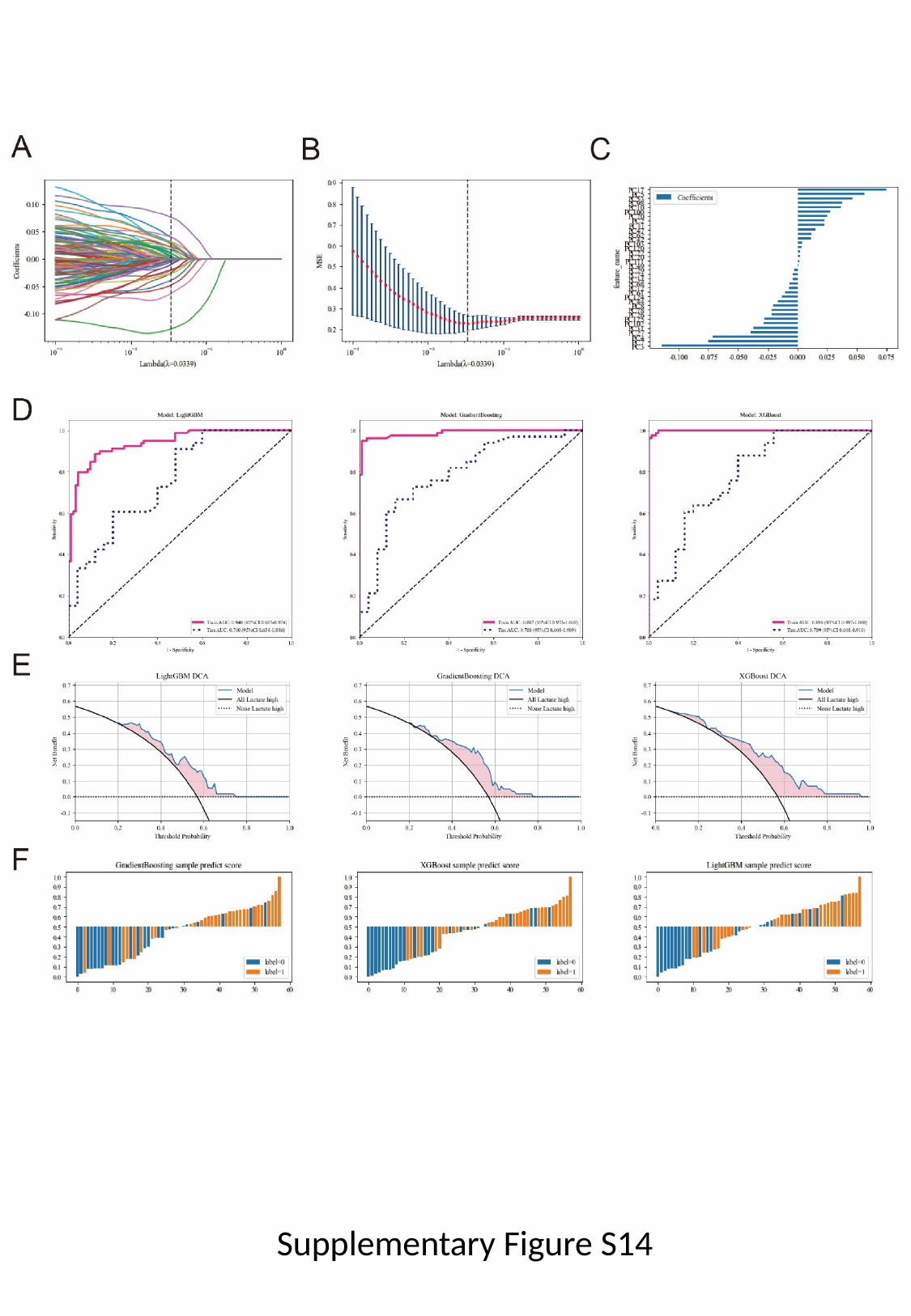

Supplementary Figure S14

## Slide 15
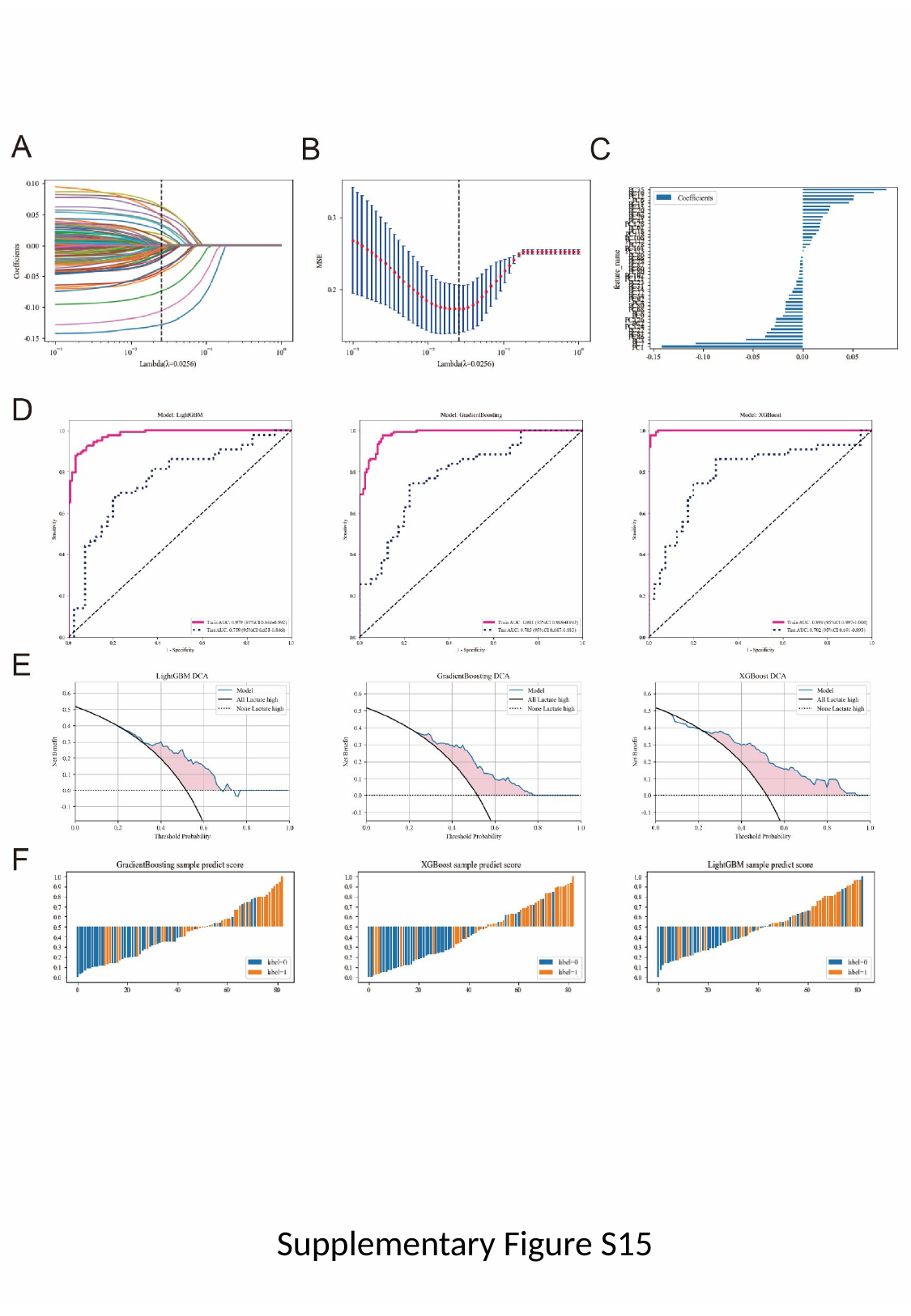

Supplementary Figure S15

## Slide 16
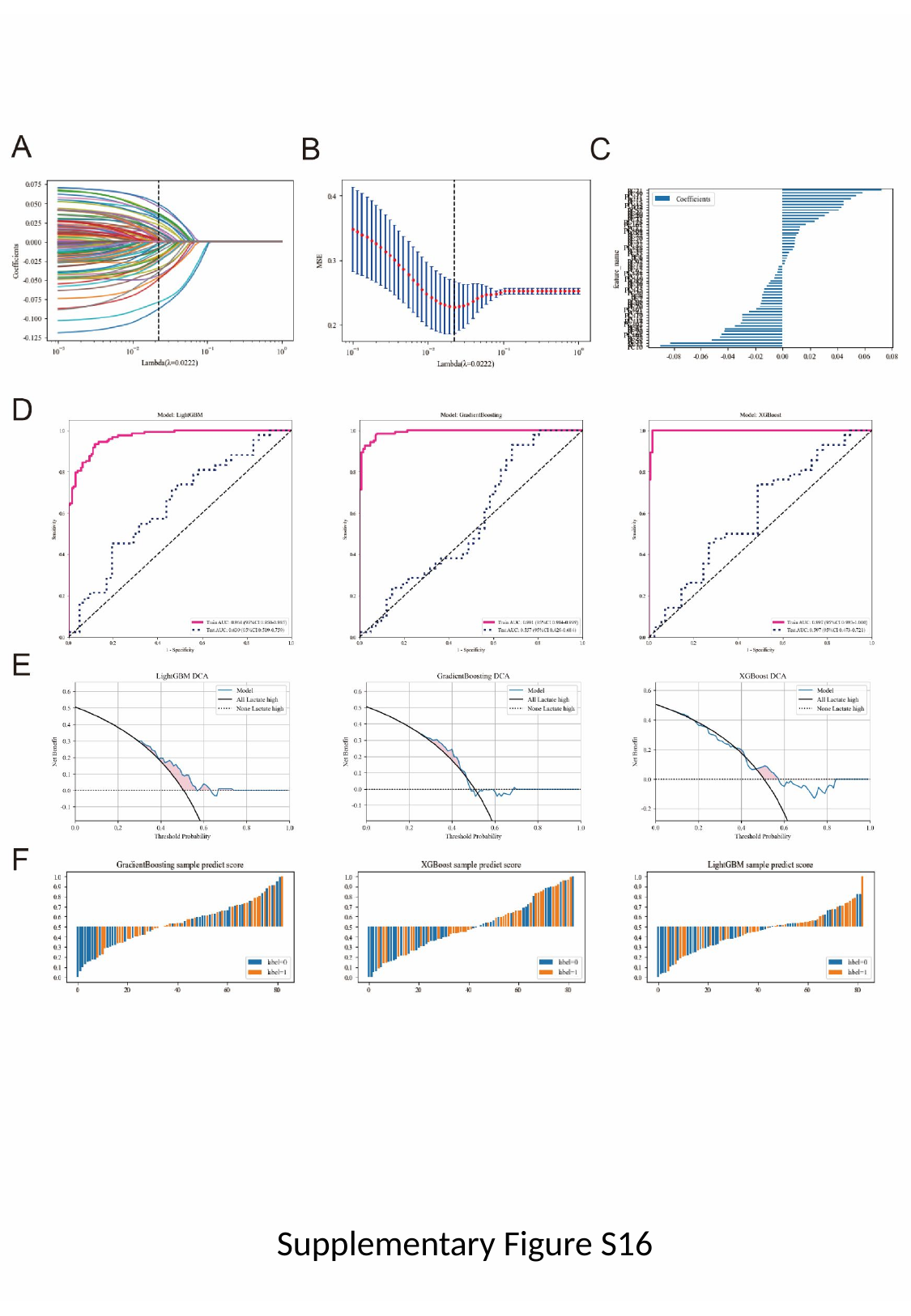

Supplementary Figure S16
